# Oxygenated phosphatidylethanolamine navigates phagocytosis of ferroptotic cells by interacting with TLR2

**DOI:** 10.1038/s41418-020-00719-2

**Published:** 2021-01-11

**Authors:** Xiang Luo, Hai-Biao Gong, Hua-Ying Gao, Yan-Ping Wu, Wan-Yang Sun, Zheng-Qiu Li, Guan Wang, Bo Liu, Lei Liang, Hiroshi Kurihara, Wen-Jun Duan, Yi-Fang Li, Rong-Rong He

**Affiliations:** 1 Guangdong Engineering Research Center of Chinese Medicine & Disease Susceptibility, Jinan University, Guangzhou, 510632 China; 2 International Cooperative Laboratory of Traditional Chinese Medicine Modernization and Innovative Drug Development of Chinese Ministry of Education (MOE), College of Pharmacy, Jinan University, Guangzhou, 510632 China; 3 Integrated Chinese and Western Medicine Department, School of Traditional Chinese Medicine, Jinan University, Guangzhou, 510632 China; 4 State Key Laboratory of Biotherapy and Cancer Center, West China Hospital, Sichuan University, Chengdu, 610041 China; 5 Collaborative Innovation Center for Biotherapy, Chengdu, 610041 China

**Keywords:** Cancer, Cancer microenvironment

## Abstract

During cancer therapy, phagocytic clearance of dead cells plays a vital role in immune homeostasis. The nonapoptotic form of cell death, ferroptosis, exhibits extraordinary potential in tumor treatment. However, the phagocytosis mechanism that regulates the engulfment of ferroptotic cells remains unclear. Here, we establish a novel pathway for phagocytic clearance of ferroptotic cells that is different from canonical mechanisms by using diverse ferroptosis models evoked by GPX4 dysfunction/deficiency. We identified the oxidized phospholipid, 1-steaoryl-2-15-HpETE-*sn*-glycero-3-phosphatidylethanolamine (SAPE-OOH), as a key eat-me signal on the ferroptotic cell surface. Enriching the plasma membrane with SAPE-OOH increased the efficiency of phagocytosis of ferroptotic cells by macrophage, a process that was suppressed by lipoprotein-associated phospholipase A_2_. Ligand fishing, lipid blotting, and cellular thermal shift assay screened and identified TLR2 as a membrane receptor that directly recognized SAPE-OOH, which was further confirmed by TLR2 inhibitors and gene silencing studies. A mouse mammary tumor model of ferroptosis verified SAPE-OOH and TLR2 as critical players in the clearance of ferroptotic cells in vivo. Taken together, this work demonstrates that SAPE-OOH on ferroptotic cell surface acts as an eat-me signal and navigates phagocytosis by targeting TLR2 on macrophages.

## Introduction

More than 100 billion short-lived or damaged cells are recycled in the human body every day. The clearance of such a remarkable mass of cell corpses, known as phagocytosis, is mainly mediated by macrophages that recognize a constellation of surface-tethered signals on dying cells [1]. Phagocytosis plays an essential role in tissue homeostasis by eliminating cells that are no longer necessary and additionally, avoiding accumulation of toxic cellular corpses [2]. In cancer therapy, recognition and removal of dying cancer cells dictate the eventual immunological consequence of the organism [3]. Given the resistance to induce apoptosis in some cancer cells [4], researchers have exploited other modes of eliminating cancer cells through nonapoptotic cell death mechanisms, such as necroptosis [5] and ferroptosis [6]. While these approaches open up new therapeutic avenues, little is known about the recognition and clearance mechanisms for cells dying through nonapoptotic cell death pathways.

Ferroptosis, a recently defined programmed cell death distinct from the classical modes, has received extensive attention particularly in the field of cancer therapy [7]. This interest comes from observations that clinically relevant tumor suppressors triggered ferroptosis, while negative regulators of ferroptosis are overexpressed or activated in a variety of tumors [8–10]. While it is recognized that many oncogenic pathways render cancer cells extremely susceptible to ferroptosis [11], there is also evidence suggesting that ferroptosis could be induced as an intrinsic antitumor mechanism that propagates through cells and tissues. This has key implications for cancer therapy since it may be possible to eliminate large groups of malignant cells in the tumor niche by triggering propagative death through ferroptosis [12]. While ferroptosis is believed to liaise closely with the immune system under various circumstances, for example, phagocytosis modulated by oxidized phospholipids (PLs) derived from ferroptotic cancer cells, this idea has not been experimentally validated [11]. Understanding the relationship between ferroptosis and phagocytosis is therefore crucial for evaluation and application of the potential of ferroptosis in cancer therapy.

To date, ferroptosis is known to be an iron-catalyzed form of regulated cell death that occurs through excessive peroxidation of polyunsaturated fatty acids (PUFAs) in plasma lipids. The precise mechanisms that trigger ferroptosis and its physiological relevance however remain elusive. Recent studies identified RSL3, an inhibitor of glutathione peroxidase-4 (GPX4) [6], as an efficient inducer of ferroptotic cell death in cancer cells [13]. This has led to the understanding that ferroptosis is largely a specialized death program caused by insufficiency of GPX4, the only known enzyme that can reduce lipid hydroperoxides within biological membranes [14–16]. Interestingly, recent redox lipidomic analyses have revealed that, out of all classes of PLs, accumulation of oxygenated arachidonoyl (AA)- or adrenoyl (AdA)-containing phosphatidylethanolamine (PE) species act as the proximate executioner of ferroptotic death [17, 18].

In this report, we used multiple ferroptosis models and determined that ferroptotic cells could be cleared by different types of macrophages. Oxygenated PE SAPE-OOH were found to be the primary phagocytic signal that promoted phagocytic clearance. We identified TLR2 as a potential candidate receptor for SAPE-OOH and established a concept of SAPE-OOH and TLR2 as critical players for engulfment of ferroptotic cells.

## Results

### Ferroptotic cells can be engulfed by macrophages

**Fig. 1:**
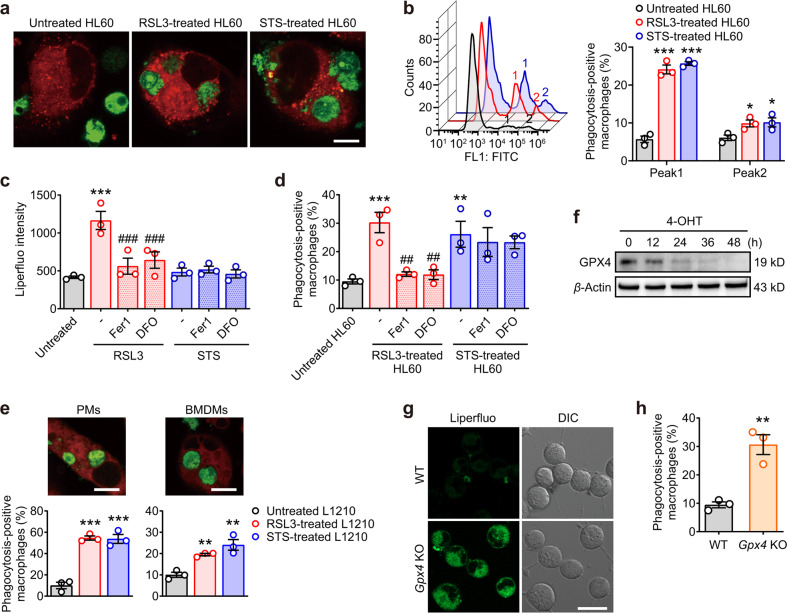
Ferroptotic cells are phagocytosed by macrophages. **a** HL60 cells (green fluorescent) were treated with RSL3 (1 μM, 6 h) or STS (0.25 μM, 6 h) and then cocultured with PMA-activated THP-1 cells (red fluorescent) for 1.5 h. The cells were imaged by confocal microscopy. Scale = 10 μM. **b** RSL3 or STS-treated HL60 cells were cocultured with PMA-activated THP-1 cells and the phagocytosis-positive macrophages was quantitatively measured by flow cytometry. “1,” Peak1, macrophages that engulfed one target cell. “2,” Peak2, macrophages that engulfed two or more target cells. **c** HL60 cells were treated with inhibitors (ferrostatin-1 (Fer1), 2 μM, 2 h; deferoxamine (DFO), 100 μM, 2 h) and RSL3/STS before staining with Liperfluo, and then the cells were measured by flow cytometry. **d** HL60 cells were treated with inhibitors Fer1 and DFO and RSL3/STS before incubation with PMA-activated THP-1 cells. Phagocytosis was measured by flow cytometry. **e** Peritoneal macrophages (PMs, left panels) or M-CSF-differentiated bone marrow-derived monocytes (BMDMs, right panels) were cocultured with RSL3-treated (5 μM, 6 h) or STS-treated (0.5 μM, 6 h) L1210 cells for 1.5 h. Phagocytosis was assessed by flow cytometry and confocal microscopy. Scale = 10 μM. **f** The protein expression of GPX4 in *Gpx4*^flox/flox^ MEFs stably expressing Cre was time-dependently decreased by 4-hydroxytamoxifen (4-OHT, 1 μM, 48 h). **g** *Gpx4* KO MEFs were stained with Liperfluo (green fluorescent) and imaged by confocal microscopy. Scale = 20 μM. **h** Primary PMs were cocultured with *Gpx4* KO MEFs for 1.5 h and the phagocytosis was measured by flow cytometry. All data represent mean ± SEM (*n* = 3 biologically independent cell cultures). ^**^P < 0.01 and ^***^P < 0.001 vs the untreated group, ^##^*P* < 0.01 and ^###^*P* < 0.001 vs the RSL3-treated group, by one-way ANOVA with LSD post hoc test (for **h**, independent samples *t*-test).

To determine the ability of macrophages to phagocytose ferroptotic cells, RSL3-treated HL60 and L1210 leukemic cells (Fig. S1a, b) were used as ferroptotic cell targets. Figure 1a and Figure S1c showed that ferroptotic HL60 cells were efficiently engulfed by THP-1-derived macrophages. Staurosporine (STS)-treated cells (Fig. S1d) were used as apoptotic cell targets for comparison (Fig. S1e). This increased clearance of target cells is due to both increased number of phagocytosis-positive macrophages and increased number of engulfed HL60 cells (Fig. 1b). To establish that ferroptosis was a form of cell death distinct from apoptosis, HL60 cells were treated with RSL3 or STS for 6 h in the absence or presence of ferroptosis inhibitors, ferrostatin-1 (Fer1) or deferoxamine (DFO). Since lipid peroxidation was well established to be a central feature of ferroptosis [13], we analyzed RSL3- and STS-treated cells for the presence of lipid peroxides using a fluorescent probe Liperfluo. An increased accumulation of oxygenated lipids in ferroptotic HL60 cells was observed, which was reversed in the presence of the ferroptosis inhibitors Fer1 or DFO. Lipid peroxides in STS-treated cells were however unaffected (Fig. 1c). These observations were in close agreement with the ability of Fer1 and DFO in inhibiting phagocytosis of RSL3-treated but not STS-treated cells (Fig. 1d). Similar results were obtained using L1210 as ferroptotic and apoptotic cell targets and primary peritoneal and bone marrow-derived macrophages (BMDMs) as the phagocytic cells (Fig. 1e).

Previous studies indicate that ferroptotic cell death occurs due to an insufficiency of the PL hydroperoxidase, GPX4 [19]. Therefore, conditional knockout of the *GPX4* gene should be expected to induce phagocytosis of ferroptotic cells by macrophages. To test this idea, we employed a 4-hydroxytamoxifen (4-OHT) inducible *Gpx4* KO model developed in *Gpx4*^flox/flox^ mouse embryonic fibroblasts (MEFs) stably expressing Cre to suppress GPX4 protein expression (*Gpx4* KO MEFs) (Fig. 1f). As shown in Fig. 1g, 4-OHT-induced GPX4 suppression coincided with increased lipid peroxidation. Assessment of phagocytosis revealed a significant increase in the clearance of *Gpx4* KO MEFs by primary macrophages when compared with WT MEFs cells (Fig. 1h). Taken together, the data presented above establish a nexus between ferroptosis, GPX4 expression, and PL peroxidation in target cells and their recognition by macrophages.

### Oxygenated PEs serve as potential phagocytic eat-me signals for the removal of ferroptotic cells

**Fig. 2:**
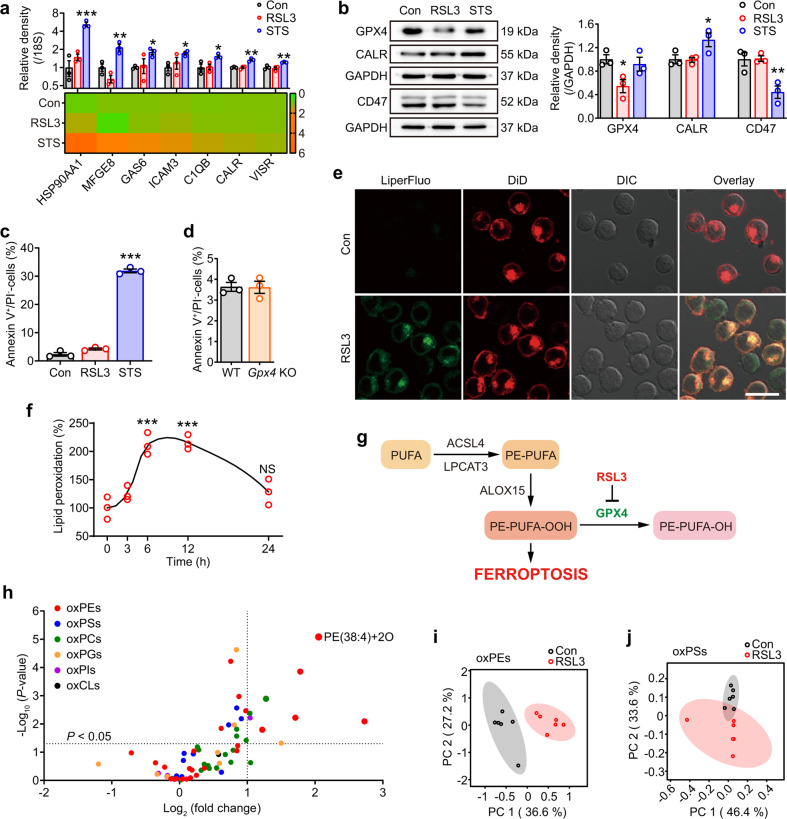
Appearance of phospholipid peroxidation products as potential phagocytic eat-me signals in ferroptotic cells. **a** HL60 cells were treated with RSL3 (1 μM, 6 h) or STS (0.25 μM, 6 h) and the gene expressions of phagocytic signals (heat shock protein HSP 90-alpha (*HSP90AA1*), milk fat globule-EGF factor 8 (*MFGE8*), growth arrest specific-6 (*GAS6*), intercellular adhesion molecule 3 (*ICAM3*), complement C1q subcomponent subunit B (*C1QB*), calreticulin (*CALR*), and V-domain Ig-containing suppressor of T-cell activation (*VISR*)) were detected by quantitative real-time PCR assay. Data are mean ± SEM (*n* = 3 biologically independent cell cultures). **b** The protein expressions of phagocytic signals (CALR and cluster of differentiation 47 (CD47)) and GPX4 in HL60 cells were determined by western blotting analysis. This experiment was repeated for three times. ^*^P < 0.05 and ^**^P < 0.01, by one-way ANOVA with LSD post hoc test. Annexin V-positive/PI-negative cells of RSL3/STS-treated HL60 cells (**c**) and *Gpx4* KO MEFs (**d**) were assessed by flow cytometry. Data are mean ± SEM (*n* = 3 biologically independent cell cultures). ^***^P < 0.001 vs the control group, by one-way ANOVA with LSD post hoc test. **e** HL60 cells were treated with RSL3 (1 μM, 6 h) and double-stained with Liperfluo (green fluorescent) and DiD (red fluorescent). Scale = 20 μM. **f** HL60 cells were treated with RSL3 (1 μM) for different hours and then cocultured with THP-1 cells. The ratio of lipid peroxidation (stained with Liperfluo) was measured by flow cytometry. ^***^P < 0.001 vs the time (0) group, by one-way ANOVA with LSD post hoc test. Data are mean ± SEM (*n* = 3 biologically independent cell cultures). **g** Scheme for simplified network of main factors regulating ferroptosis. PUFA polyunsaturated fatty acids, PE phosphatidylethanolamines. **h** HL60 cells were treated with RSL3 (1 μM, 6 h) before the plasma membrane was isolated and detected by LC–MS/MS-based phospholipidomics. Data are mean ± SEM; *n* = 6 biologically independent cell cultures. Obtained data were displayed as volcano plots showing the changes in the levels of oxygenated phospholipids (log_2_ (fold change), *X*-axis) vs significance (−log_10_ (*P* value), *Y*-axis, by *t*-test). ox oxygenated. Each dot represents one class of phospholipids. Data of oxPEs (**i**) and oxPSs (**j**) were extracted and interpreted by principal component analysis (PCA), and the 2D score plots displayed repertoires of control and RSL3-treated cells. Ellipses: 95% confidence regions.

Apoptotic cells are known to present a variety of cell surface signals that trigger their recognition by macrophages [1]. For example, HL60 cells induced to undergo apoptosis with STS showed a significant increase in expression of eat-me signals compared to the untreated control (Fig. 2a, b). Induction of ferroptosis in these cells using RSL3 however did not promote expression of these signals (Fig. 2a, b). Specifically, the expression levels of the eat-me signal calreticulin and the don’t eat-me signal CD47 were altered in STS-treated cells, but not in the RSL3-treated cells when compared to untreated controls (Fig. 2b).

It is well established that PLs participate in the signaling of cellular interactions, and the translocation of phosphatidylserine (PS)/oxygenated PS (oxPS) from the inner to the outer leaflet of the lipid bilayer are characterized to be fundamental and effective eat-me signals in the phagocytosis of apoptotic cells [20, 21]. The exposure of PS occurs very early during the apoptotic process [21], consistent with the findings of annexin V positivity that appeared much earlier than the time of PI positivity in apoptotic cells (Fig. S2). However, data showed that annexin V-positive ferroptotic cells were not significantly increased (Fig. 2c, d) until there is an increase in PI staining (Fig. S2), suggesting that PS is not presented on the outer surface of plasma membrane when ferroptotic cells initiate phagocytosis. Taken together, these results suggest that canonical eat-me signals relevant to apoptosis are likely irrelevant to the clearance of ferroptotic cells.

It was noteworthy in these experiments that expression of GPX4 in HL60 cells was not affected by STS treatment but reduced significantly following RSL3 treatment [22, 23] (Fig. 2b). Given that GPX4 is the only known enzyme that can effectively reduce PL peroxide to the corresponding alcohols [17] and we observate that *Gpx4* KO cells accumulated lipid peroxidation products (Fig. 1g), we hypothesize that peroxidized lipids are likely an eat-me signal that promotes elimination of ferroptotic cells by phagocytes. This hypothesis was supported by the observation that RSL3 treatment resulted in the appearance of lipid peroxidation products (green fluorescence) that colocalized with cellular membranes (DiD-positive), including the plasma membrane (red fluorescence) (Fig. 2e). A time-dependent RSL3-treatment experiment yielded a sigmoidal curve showing a sudden rise in lipid peroxidation at 6 h, which lasted up to 12 h (Fig. 2f). Importantly these results were coincident with the time-dependent phagocytosis data described earlier (Fig. S1e). Lipid peroxidation products dwindled at 24 h, likely due to truncation of lipid products and severe disruption of plasma membrane [24, 25] (Fig. 2f). To verify whether the eat-me signal of ferroptotic cells is associated with the esterified oxygenated PUFA on the cell surface, we enriched expression of unsaturated PLs in HL60 cells by supplementing the growth medium with exogenous arachidonic acid (AA). Culturing HL60 cells in the presence of AA resulted in an enrichment of unsaturated fatty acids in all PL classes (Fig. S6a). Exogenous AA promoted the phagocytosis of target cells in a dose-dependent manner (Fig. S6b), and exhibited even higher efficiency on phagocytosis after RSL3 treatment (Fig. S6c), underscoring the relevance between the degree of PL unsaturation and the clearance of ferroptotic cells.

To verify the above inference, we applied phospholipidomics based on liquid chromatography–mass spectrometry (LC–MS/MS) analysis to identify six major classes of PLs—PE, phosphatidylcholine, phosphatidylinositol, PS, phosphatidylglycerol, and cardiolipin in naive HL60 cells (Fig. S3a). Incubating these cells with RSL3 to induce ferroptosis resulted in the appearance of oxygenated PLs (oxPLs) across all species (Fig. S3b). It is well accepted that ferroptosis is marked by oxidative modification of PUFAs containing membrane PLs, especially the conversion of PE to its hydroperoxides [11, 17, 19] (Fig. 2g). The plasma membrane was further isolated and identified with multiple biofilm markers (Fig. S4a), based on the consideration that as eat-me signals, molecules must be present on the cell surface. Among all classes of oxPLs detected (Fig. S4b), the oxPEs were the most predominant species with significant fold change (Fig. 2h), and SAPE-OOH (PE(38:4)+2O) showed the most reliable increase when compared to naive cells (Fig. 2h). Besides, analysis of singly, doubly, and triply oxPLs demonstrated that hydroperoxides were the major form of oxPLs in ferroptotic cells (Fig. S4c). Principal component analysis (PCA) revealed that, compared with oxPSs, the input feature of oxPEs displayed much more distinct repertoires from control and RSL3-treated cells (Fig. 2i, j). Taken together, present results suggest that canonical eat-me signals relevant to apoptosis are likely irrelevant to the clearance of ferroptotic cells. PE hydroperoxides, particularly the SAPE-OOH, seem to prevail substantially on the surface of ferroptotic cells and act potentially as a phagocytic signal.

### PE hydroperoxides (SAPE-OOH) provide the eat-me signal for phagocytosis of ferroptotic cells

**Fig. 3:**
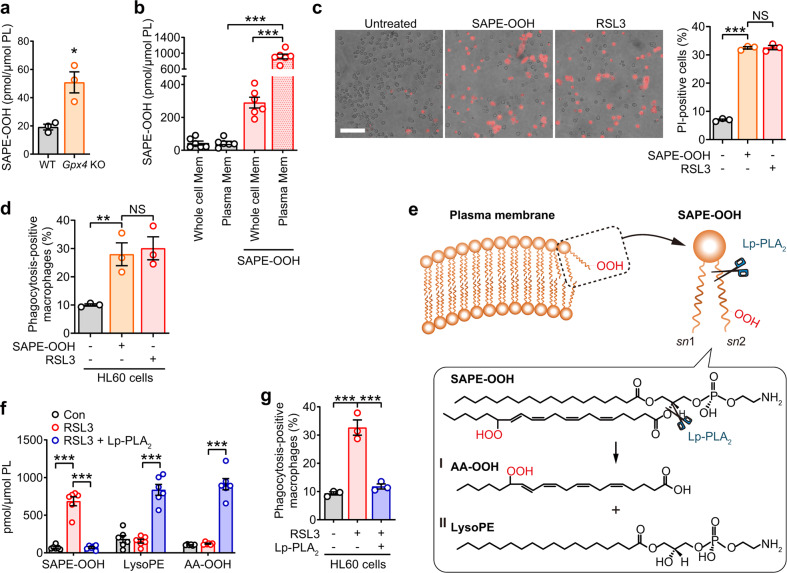
SAPE-OOH acts as a specific signal in GPX4-deficiency-induced ferroptosis. **a** Content of SAPE-OOH was determined by LC–MS/MS in *Gpx4* KO MEFs. Data are mean ± SEM (*n* = 3 independent biologically cell cultures). **b** HL60 cells were treated with SAPE-OOH (2.5 μM, 6 h) before the plasma membrane was isolated. Content of SAPE-OOH was assessed by LC–MS/MS. Mem membrane. Data are mean ± SEM (*n* = 6 independent biologically cell cultures). **c** PI staining was used to conduct and to examine the ferroptosis in HL60 cells treated with SAPE-OOH (2.5 μM, 6 h) and RSL3 (1 μM, 6 h). Scale = 100 μM. Data are mean ± SEM (*n* = 3 independent biologically cell cultures). **d** Phagocytosis of THP-1 cells to HL60 cells treated with SAPE-OOH (2.5 μM, 6 h) or RSL3 (1 μM, 6 h). Data are mean ± SEM (*n* = 3 independent biologically cell cultures). **e** Scheme for Lp-PLA_2_ hydrolyzing membrane SAPE-OOH into (I) AA-OOH and (II) lysoPE. **f** HL60 cells were treated with RSL3 and Lp-PLA_2_ (2.5 μg/10^6^-cells) for 1 h. AA-OOH and lysoPE content were determined by LC–MS/MS. Data are mean ± SEM (*n* = 6 independent biologically cell cultures). **g** Phagocytosis of THP-1 cells to HL60 cells that were treated with RSL3 and Lp-PLA_2_. Data are mean ± SEM (*n* = 3 independent biologically cell cultures). ^*^P < 0.05, ^**^P < 0.01 and ^***^P < 0.001, by one-way ANOVA with LSD post hoc test (for **a**, independent samples *t*-test).

Same as above, SAPE-OOH, confirmed as 1-stearoyl-2-15-HpETE-*sn*-glycero-3-phosphatidylethanolamine (Fig. S5a), was predominantly present in AA-enriched ferroptotic cells compared to naive HL60 cells (Fig. S6d), as well as in *Gpx4* KO MEFs compared to their WT counterparts (Fig. 3a). These results are also in agreement with recent reports identifying SAPE-OOH as a specific ferroptotic death signal [26]. From this, naive HL60 cells were supplemented with SAPE-OOH to evaluate its role of inducing phagocytosis. Although exogenously added PLs could be effectively internalized by translocases, we found that SAPE-OOH was primarily located on the plasma membrane compared to the biofilm of the whole cell (Fig. 3b). Importantly, naive HL60 cells supplemented with SAPE-OOH were equally effective targets for ferroptosis (Fig. 3c) and phagocytosis by macrophages (Fig. 3d) as those induced by RSL3. The relevance of SAPE-OOH as a critical eat-me signal for ferroptotic cell clearance was further confirmed by pretreating the RSL3-incubated cells with lipoprotein-associated phospholipase A_2_ (Lp-PLA_2_) before the assessment of phagocytosis. LC–MS/MS-based fragmentation analysis (Fig. S5) verified that Lp-PLA_2_ treatment, which was shown to preferentially hydrolyze oxidatively modified PLs (Fig. 3e) [27], resulted in a decrease in SAPE-OOH due to its hydrolysis at the *sn*-2 acyl bond of PLs yielding AA-OOH and 1-stearoyl-2-OH-*sn*-glycero-3-phosphatidylethanolamine (lysoPE) (Fig. 3f), which correlated with significantly reduced clearance by macrophages (Fig. 3g). These experiments establish the importance of PE hydroperoxides rather than PEs, lysoPE or free fatty acid hydroperoxides as an engulfment signal for clearance of ferroptotic cells.

### Macrophage TLR2 mediates recognition of SAPE-OOH on the ferroptotic cell surface

**Fig. 4:**
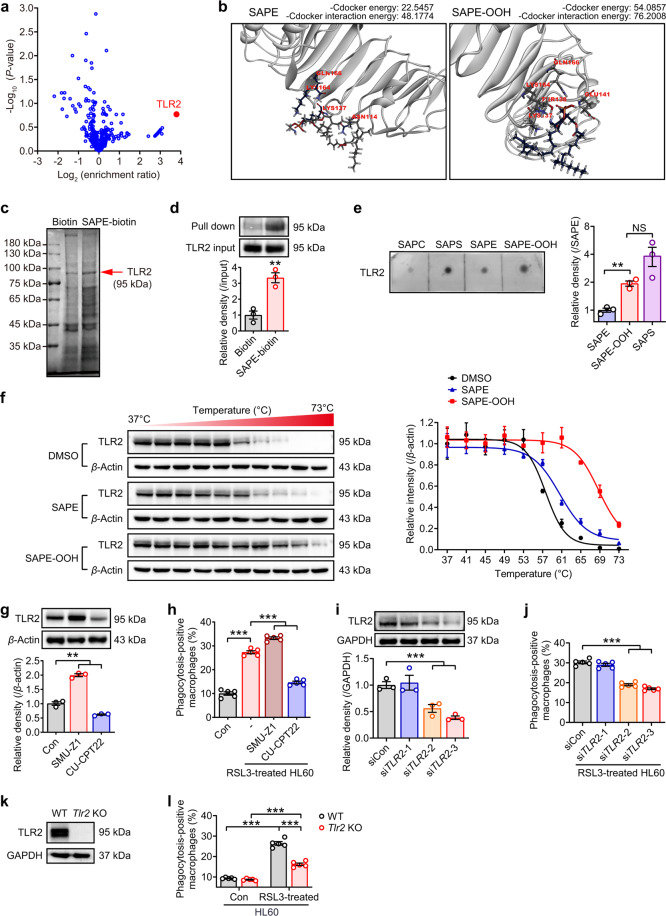
TLR2 is a potential phagocytosis receptor for SAPE-OOH. **a** Identification of SAPE binding proteins by LC–MS/MS-based shotgun proteomics. Volcano plots presented the ratio of label-free quantification (LFQ, log_2_ (enrichment ratio), *X*-axis) vs significance (−log_10_ (*P* value), *Y*-axis, by *t*-test) of SAPE-biotin and biotin group. Data are mean ± SEM (*n* = 3 independent biologically cell cultures). **b** Molecular docking pattern of SAPE and SAPE-OOH with TLR2. **c** Pull-down assay using the lysates of THP-1 cells that were coincubated with RSL3-treated (1 μM, 6 h) HL60 cells. The gels were stained with Coomassie Brilliant Blue. **d** Western blotting-based pull-down assay was conducted to detect the combination of TLR2 to SAPE-biotin. Data are mean ± SEM (*n* = 3 independent biologically cell cultures). **e** Dot-blot binding approach of phospholipids with TLR2. Immobilized lipids were incubated with recombinant TLR2 protein. Binding was visualized using HRP-conjugated antibody. Data are mean ± SEM (*n* = 3 independent biologically cell cultures). **f** Thermal stabilization of TLR2 in THP-1 cells incubated with DMSO, SAPE (2.5 μM, 1 h), and SAPE-OOH (2.5 μM, 1 h) were determined following standard cellular thermal shift protocol with heat treatment from 37 to 73 °C. Data are mean ± SEM (*n* = 3 independent biologically cell cultures). **g** Western blotting of TLR2 expression in THP-1 cells treated with SMU-Z1 (1 μM) and CU-CPT22 (10 μM) for 24 h. Data are mean ± SEM (*n* = 3 independent biologically cell cultures). **h** Phagocytosis of indicated reagents-treated THP-1 cells to ferroptotic HL60 cells. Data are mean ± SEM (*n* = 5 independent biologically cell cultures). **i** Western blotting of TLR2 expression in THP-1 cells treated with three different si*TLR2*s. Data are mean ± SEM (*n* = 3 independent biologically cell cultures). **j** Phagocytosis of si*TLR2*s-treated THP-1 cells to ferroptotic HL60 cells. Data are mean ± SEM (*n* = 5 independent biologically cell cultures). **k** Western blotting of TLR2 expression in murine primary PMs derived from *Tlr2* KO mice. **l** Phagocytosis of murine primary PMs derived from *Tlr2* KO mice to ferroptotic HL60 cells. Data are mean ± SEM (*n* = 3 independent biologically animals). ^**^P < 0.01 and ^***^P < 0.001, by one-way ANOVA with LSD post hoc test (for **d** and **e**, independent samples *t*-test).

To identify the macrophage receptors that interact with SAPE-OOH on ferroptotic cells, we performed receptor-based ligand fishing experiments. Biotinylated SAPE (SAPE-biotin) was synthesized (Fig. S7 and S8) and incubated with PMA-activated THP-1 cells that were exposed to RSL3-induced ferroptotic HL60 cells to identify potential cellular targets of SAPE by pull-down assay/LC–MS/MS. As a result, TLR2 was identified as the most increased protein hit in the SAPE-treated sample vs negative control (log_2_ (enrichment ratio) > 2.0) (Fig. 4a), indicating that SAPE could interact with TLR2 in cellular conditions. Furthermore, we carried out molecular docking analysis between TLR2 and SAPE or SAPE-OOH. TLR2 was predicted to highly and directly interact with both species and have greater specificity to SAPE-OOH according to the binding scores (Fig. 4b). Taken together, we speculate TLR2 to be the potential candidate receptor for SAPE-OOH.

To ascertain this assumption, we lysed THP-1 cells after coincubating with ferroptotic cells and analyzed the proteins combined with SAPE-biotin by gel electrophoresis-based pull-down assay. The ≈95 kDa protein (appeared to be TLR2) was obviously denser in the SAPE-biotin lane than in the biotin only control lane (Fig. 4c). In addition, by applying TLR2 antibody, SAPE-biotin was shown to be a much more attractive target than biotin for TLR2 (Fig. 4d). Furthermore, we performed dot-blot binding analysis utilizing recombinant TLR2 to evaluate its ability to bind SAPE and SAPE-OOH. PS, an established TLR2 ligand [28] and a PL that was irrelevant in ferroptosis (Fig. 2c, d and Fig. S2), was used as a positive control in the binding analysis. As seen in Fig. 4e, a preferential binding of TLR2 to SAPE-OOH was observed. Cellular thermal shift assay (CETSA) was conducted to provide direct evidence for the SAPE-OOH–TLR2 interaction. Compared with control and SAPE groups, SAPE-OOH significantly increased the thermal stabilization (Fig. 4f), suggesting a preferentially higher binding of SAPE-OOH with TLR2 protein.

To establish the relevance of TLR2 in clearance of ferroptotic cells, macrophages were pretreated with the pharmacological TLR2 agonist SMU-Z1 [29] or TLR2 antagonist CU-CPT22 [30] to alter protein expression (Fig. 4g) prior to the phagocytosis assay. Figure 4h showed that the clearance of ferroptotic cells was significantly enhanced by SMU-Z1 and conversely, significantly inhibited by CU-CPT22. Unequivocal confirmation of a role for TLR2 was obtained since SMU-Z1 and CU-CPT22 also affected TLR1 responses, unequivocal confirmation of a role for TLR2 in SAPE-OOH dependent clearance of ferroptotic cells was obtained using siRNA. We observed that the reduction in TLR2 expression level in siRNA-treated macrophages (Fig. 4i) mirrored a reduction in phagocytosis-positive macrophages (Fig. 4j) demonstrating a direct role for TLR2 in phagocytosis of ferroptotic cells. Confirmation of TLR2 as a key receptor for SAPE-OOH-dependent phagocytosis of ferroptotic cells was obtained using murine primary macrophages from *Tlr2* KO mice (Fig. 4k), which exhibited significantly reduced phagocytosis of RSL3-treated HL60 cells (Fig. 4l) in macrophages that did not express TLR2, compared to WT control cells. It was noteworthy that phagocytosis in *Tlr2* KO macrophages did not reduce to the same levels as naive untreated HL60 cells (Fig. 4l), indicating the presence of compensatory mechanisms whereby other macrophage receptors took over the job of clearing ferroptotic cells when TLR2 receptors were unavailable.

### SAPE-OOH and TLR2 are critical players for clearance of ferroptotic cells in vivo

**Fig. 5:**
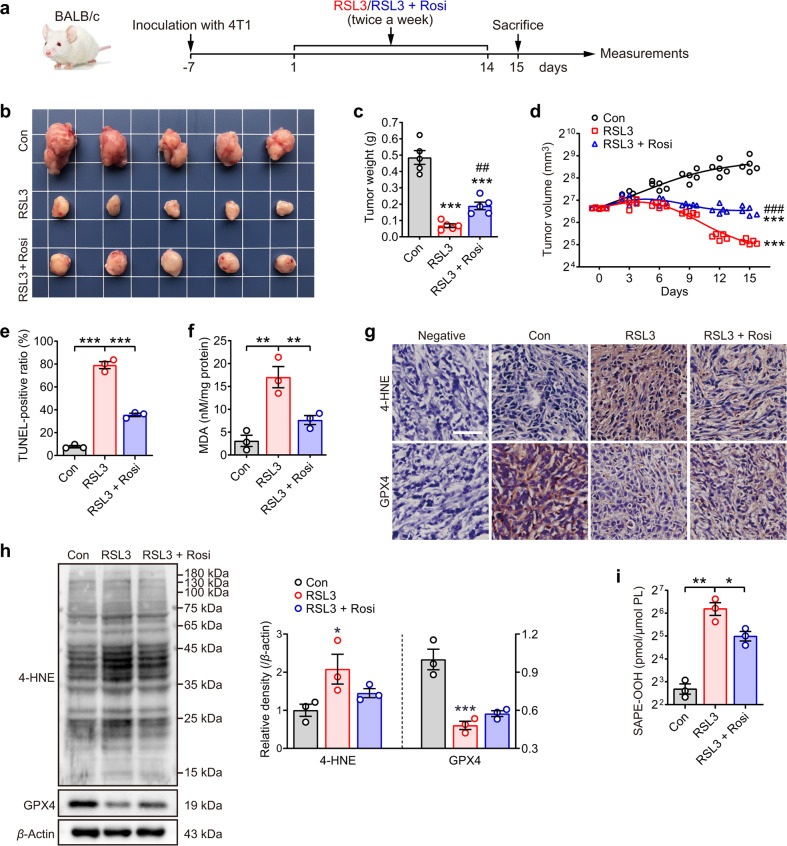
Ferroptosis and lipid peroxidation are induced in mammary tumor-bearing mice. **a** Female BALB/c mice carrying the 4T1 cell-formed tumors at the mammary fat pads were treated with RSL3 alone or combined with rosiglitazone (Rosi). Tumor growth in mice was presented by images (**b**), as well as assessed by tumor weight (**c**, ^***^P < 0.001 vs the control group and ^##^*P* < 0.01 vs the RSL3 group, by independent samples *t*-test, respectively) and tumor volume (**d**, ^***^P < 0.001 vs the control group and ^###^*P* < 0.001 vs the RSL3 group, by two-way repeated measures AVONA). Data are mean ± SEM (*n* = 5 independent biologically animals). **e** TUNEL assay was carried out to evaluate the death ratio of tumor cells. Data are mean ± SEM (*n* = 3 independent biologically animals). **f** Tumor cells were measured by MDA assay. Data are mean ± SEM (*n* = 3 independent biologically animals). ^**^P < 0.01 and ^***^P < 0.001, by one-way ANOVA with LSD post hoc test. **g** Immunohistochemistry of tumor sections labeled with antibodies of 4-HNE and GPX4. Scale = 50 μM. **h** Western blotting analysis of 4-HNE and GPX4 expression in tumor. Each immunoblot represents three biological repeats, and representative blotting results are shown. **i** Content of SAPE-OOH in tumor cells was determined by LC–MS/MS. Data are mean ± SEM (*n* = 3 independent biologically animals). ^*^P < 0.05, ^**^P < 0.01 and ^***^P < 0.001 by one-way ANOVA with LSD post hoc test. All data represent mean ± SEM.

To confirm the stimulatory effect of SAPE-OOH on phagocytosis in vivo, a mouse tumor model was employed. Female BALB/c mice were injected with 4T1 mammary carcinoma cells in the mammary fat pads. After palpable tumors were established (~100 mm^3^), the mice were divided into three groups to receive saline (control), RSL3, or RSL3 coadministered with the acyl-CoA synthetase (ACSL4) inhibitor rosiglitazone (Rosi) as shown in Fig. 5a. No substantial changes in body weight of the mice enduring these treatments (Fig. S9a) were observed. At day 15, the animals were sacrificed and the tumors were resected and weighed. A significant reduction in tumor weight and volume was observed in the RSL3-treatment group, which was partly reversed in the RSL3 + rosiglitazone group [31] (Fig. 5b–d). TUNEL staining revealed that RSL3 resulted in significant cell death of tumor cells, which was moderated by rosiglitazone treatment (Fig. 5e and Fig. S9b). Further analysis of tissue sections for malondialdehyde (MDA) levels showed RSL3-increased cellular lipid peroxides in tumor, which was attenuated when rosiglitazone was coadministered (Fig. 5f). In agreement with recent studies from other groups [32, 33], we also showed an increase in 4-HNE expression and a decrease in GPX4 expression when mice were treated with RSL3 (Fig. 5g, h). Besides, these data were consistent with the LC–MS/MS data, showing that RSL3 treatment induced an increase in SAPE-OOH, which declined in the coadministered group (Fig. 5i).

**Fig. 6:**
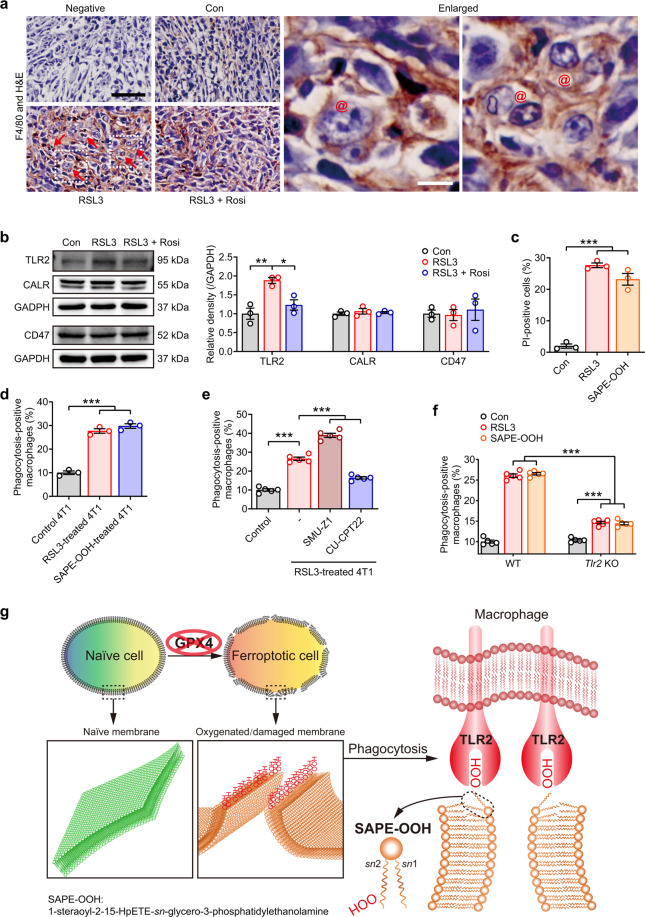
The phagocytosis of ferroptotic tumor cells is dependent on the interaction of SAPE-OOH and TLR2. **a** The phagocytosis was observed by immunohistochemistry of F4/80-labeled and  H&E stained tumor sections. Red arrows: F4/80-positive cells. @: macrophages containing multiple nuclei. Enlarged: images in dashed frames. Scale = 100 μM (black) and 10 μM (white). **b** The protein expressions of the indicated proteins in tumor tissues. Each immunoblot represents three biological animals, and representative blotting results are shown. **c** PI staining of 4T1 cells treated with RSL3 (1 μM, 6 h) or SAPE-OOH (2.5 μM, 6 h). Data are mean ± SEM (*n* = 3 independent biologically cell cultures). **d** Phagocytosis of 4T1 cells treated with RSL3 or SAPE-OOH. Data are mean ± SEM (*n* = 3 independent biologically cell cultures). **e** THP-1 cells pretreated with SMU-Z1 (1 μM) and CU-CPT22 (10 μM) for 24 h. Phagocytosis of 4T1 cells treated with RSL3. Data are mean ± SEM (*n* = 5 independent biologically cell cultures). **f** L1210 cells (previously treated with RSL3 (5 μM, 6 h) or SAPE-OOH (10 μM, 6 h) were injected into the peritoneum of WT or *Tlr2* KO mice. Total peritoneal cells were harvested and labeled with F4/80 before the in vivo phagocytosis was assessed by flow cytometry. Data are mean ± SEM (*n* = 5 independent biologically cell cultures). **g** Graphic abstract: SAPE-OOH on ferroptotic cell surfaces acts as an eat-me signal and navigates phagocytosis by targeting TLR2 on macrophages. All data represent mean ± SEM. ^*^P < 0.05, ^**^P < 0.01 and ^***^P < 0.001, by one-way ANOVA with LSD post hoc test.

Induction of ferroptosis in the 4T1 tumors by RSL3 administration is expected to activate the homeostatic machinery leading to infiltration of macrophages. Indeed, F4/80 staining showed a significantly higher infiltration rate of macrophages in tumors treated with RSL3, but not the saline controls (Fig. 6a). A higher magnification image of the tissue sections revealed that ferroptotic cells were visibly engulfed by macrophages in the tumor tissue from mice treated with RSL3 alone, but not the coadministered group (Fig. 6a). Since rosiglitazone is known to suppress RSL3-induced production of oxidized PEs (23), the tumor data described above illustrate the crucial requirement of oxidized PE in the clearance of ferroptotic cells by macrophages. Furthermore, as observed for HL60 cells (Fig. 2a, b), RSL3 was able to promote engulfment of ferroptotic cells by macrophages without affecting CALR and CD47 expression (Fig. 6b). On the other hand, RSL3 induced significant upregulation of TLR2 expression in tumor tissues, which was reversed by the inclusion of rosiglitazone (Fig. 6b).

An in vitro 4T1 mammary carcinoma cell model was used to verify TLR2 as a ubiquitous receptor that piloted phagocytosis of ferroptotic cells. As observed with HL60 cells, RSL3 and SAPE-OOH treatment triggered ferroptosis of 4T1 cells (Fig. 6c). Ferroptotic 4T1 cells were efficiently engulfed by THP-1-derived macrophages (Fig. 6d). Similarly, SMU-Z1 and CU-CPT22 significantly increased and decreased, respectively, the efficiency of phagocytosis of these cells by macrophages (Fig. 6e). Furthermore, the role of SAPE-OOH involved in TLR2 signaling was confirmed through evaluating the in vivo phagocytic capacity of L1210 cells injected intraperitoneally in *Tlr2* KO mice. Consistent with the data of *Tlr2* KO in vitro (Fig. 4l), *Tlr2* KO mice showed significantly decreased phagocytosis upon the stimulation of RSL3 and SAPE-OOH compared to WT mice, although the engulfment of ferroptotic cells was not completely abolished by TLR2 absence (Fig. 6f). In conclusion, the animal experiments together with the in vitro experiments establish a role for PL peroxidation and specifically, SAPE-OOH in TLR2 dependent clearance of ferroptotic cells.

## Discussion

In the current study, we explored the critical players that facilitate recognition and clearance of ferroptotic cells by macrophages. We determined that ferroptotic cells were readily engulfed by different types of macrophages through a mechanism different from the engulfment of apoptotic cells. Using LC–MS/MS-based phospholipidomics, we found that phagocytosis of ferroptotic cells occurred simultaneously with the accumulation of oxPLs, especially PE hydroperoxides. Through further phospholipidomics analysis of the plasma membrane of ferroptotic cells, we established that SAPE-OOH is the primary eat-me signal in ferroptotic cells that promoted phagocytic clearance. Hence, we employed a combination of shotgun proteomics analysis, lipid blotting, and CETSA approaches to identify the macrophage TLR2 receptor as the factor that was responsible for directly recognizing SAPE-OOH. Subsequent verification of the role for SAPE-OOH–TLR2 interaction in triggering phagocytosis of ferroptotic cells was established by experiments applying TLR2 inhibitors, *Tlr2* silencing, and *Tlr2* KO transgenic mice. Taken together, the data establish the concept that the SAPE-OOH navigates the phagocytosis of ferroptotic cells by directly interacting with TLR2 (Fig. 6g).

Phagocytosis is a crucial mechanism that maintains tissue homeostasis by removing damaged, dying, or effete cells, which may be toxic to the body [1]. Efficient phagocytosis requires the presentation of specific eat-me signals on the surface of dying cells. While the ligands and receptors that mediate clearance of apoptotic cells are well established, those that dictate the removal of cells dying from other forms of cell death especially ferroptosis remain ambiguous. Using a well-established RSL3-induced ferroptosis model, we show here that phagocytosis of ferroptotic cells differs on many aspects from that of apoptotic cells. RSL3-dependent ferroptosis occurs through the depletion of the PL-specific hydroperoxidase GPX4 and that conditional knockout of *GPX4* replicates many of the characteristics of ferroptotic cells including SAPE-OOH and enhanced phagocytosis. We also showed that ferroptotic cells were engulfed as effectively as apoptotic cells. A recent publication suggests that ferroptotic cell clearance is less effective than that of apoptotic cells [34], which is different from our observation. This discrepancy could be due to the use of a different cell line (Jurkat in previous publication) [34]. Here, we demonstrated consistent results obtained from a range of cell lines including HL60 cells, MEFs, and 4T1 cells as the ferroptotic cell death models, and primary as well as immortalized macrophages as the phagocyte model. Furthermore, our in vivo data highlighted the notion that ferroptotic cancer cells could be efficiently eliminated through phagocytosis.

It is presently believed that ferroptotic cancer cells interact with the immune system through a process that could potentially be modulated by oxidized PLs, though this requires experimental validation [11]. In this regard, cells in which PLs were enriched with the SAPE-OOH or AA by metabolic methods were engulfed better in vitro after induction of ferroptosis. Conversely, rosiglitazone, an inhibitor of ACSL4 which catalyzes PUFA incorporation into PLs, mitigated the in vivo elimination of tumor cells in RSL3-administrated mice. These observations on phagocytosis directly correlate with the increase in SAPE-OOH upon induction of ferroptosis, which is reversed in the inclusion of rosiglitazone. Our data therefore establish a nexus between SAPE-OOH, ferroptosis, and formation of PE hydroperoxides, whereby the cells are primed for detection and elimination by the phagocytic machinery that is a component of the immune system. Moreover, as a marker of cells undergo ferroptosis [17, 18], PE hydroperoxides have also been reported to be generated via the lipoxygenase ALOX15, an enzyme that is also believed to participate in the release of immunomodulatory signals that affect DCs maturation [35]. These reports are interesting and appear to reconcile with our observations on SAPE-OOH as modulators of ferroptosis cell clearance. Our observations may help to establish a common concept as to the role for oxidized PS in the efficient clearance of apoptotic cells [20].

Proteomics, lipid blotting, and CETSA analysis identified TLR2 as the macrophage receptor that enabled SAPE-OOH triggered clearance of ferroptotic cells. While TLR2 is also known to bind oxPSs [28], the PCA plots obtained from phospholipidomics indicated that the feature of oxPEs displayed much more distinct repertoires than oxPSs. In conclusion, we demonstrate that ferroptotic cells are efficiently engulfed by macrophages through a mechanism that requires the interaction between SAPE-OOH on ferroptotic cells and TLR2 on phagocytes. Nonetheless, we found that even if the *Tlr2* gene was knocked out, the engulfment of ferroptotic cells was still remarkably stronger than that of naive cells. This assessment suggests that, apart from the SAPE-OOH–TLR2 pathway, there must be other signals responsible for this process, although additional studies are required to substantiate this idea. On the other hand, although PE is the most significantly and universally oxPL class during ferroptosis, the involvement of other oxidized PL classes (e.g., SAPC-OOH) need to be examined to complete the ferroptotic cell-related phagocytosis theory. These processes may have implications in immunity in general and the anticancer immunity in particular. While there are reports showing that the DAMP, HMGB1 is released by ferroptotic cells [36] and this factor might support ferroptosis as immunogenic in nature similarly to necroptosis, additional investigation is warranted to understand the relevance of ferroptotic cell clearance vis-a-vis clearance of apoptotic and necrotic cells in the balance between proinflammatory and anti-inflammatory responses in normal physiology and pathophysiology in diseases including cancer.

As mentioned earlier, PSs and oxPSs are reported to be fundamental and effective eat-me signals participating in the phagocytosis of apoptotic cells. However, we believe that the ferroptosis-associated eat-me signal is more likely to be oxPEs based on a number of lines of evidence presented in the current study. Subsequent experiments demonstrated that SAPE-OOH might act as a ferroptosis-associated eat-me signal. Nevertheless, at this moment, we can only propose the idea that SAPE-OOH functions as one of the ferroptosis-related eat-me signals, probably due to its ubiquitously existence in the plasma membrane. Whether oxPSs is also involved in the phagocytosis signaling during the late stage of ferroptosis remains to be further investigated in future studies.

## Methods

### Experimental design

Emerging studies show the potential of ferroptosis triggering for cancer therapy [7]. Although a lot of effort has been spent on designing and developing anticancer drugs based on ferroptosis induction, very little is known about recognition and clearance mechanisms for ferroptotic cells. Here, we are aiming to illustrate the relationship between ferroptosis and phagocytosis, which is crucial for evaluation and application of the anticancer potential of ferroptosis.

In current study, we used multiple ferroptosis models to determine whether ferroptotic cells could be engulfed by different types of macrophages. We explored mechanisms of evoking phagocytosis by PCR, western blot, and flow cytometry detecting canonical signals and reported specific ferroptotic signals. Using LC–MS/MS, oxygenated PE SAPE-OOH was found to be the primary phagocytic signal that promoted phagocytic clearance. Both increasing and decreasing the SAPE-OOH in plasma membrane affected the phagocytosis rate toward ferroptotic cells. We employed ligand fishing, lipid blotting, and CETSA analysis to screen and identify TLR2 as a membrane receptor that recognizes SAPE-OOH, which was further confirmed by experiments utilizing TLR2 inhibitors, *Tlr2* silencing, and *Tlr2* KO mice. A mouse mammary tumor model of ferroptosis verified SAPE-OOH and TLR2 as critical players in the clearance of ferroptotic cells in vivo. Taken together, this work demonstrates that the SAPE-OOH on ferroptotic cell surfaces acts as an eat-me signal and navigates phagocytosis by targeting TLR2 on macrophages.

The sample sizes of each set of cells/animals were determined as at least three biologically independent cell cultures for in vitro experiments and five biologically independent animals for in vivo experiments, according to previous studies performed by our group. Particularly, six biologically independent cell cultures were performed for LC–MS/MS analysis, and three biologically independent animals were performed for western blot assay. At least three biological replicates were performed for each experiment and each experimental group/condition of sample size was shown in figure legend. No exclusions of data were made that would significantly impact the results or conclusions. The cell lines/animals with the same genotype and similar baseline values were randomly assigned to treated groups. Western blot and qPCR data were grouped, but the samples were loaded randomly. The investigators were blinded regarding treatment or genotype during experimental procedures or data acquisition.

### Cell lines

THP-1 cells (Wuhan University Cell Bank, Wuhan, China) were maintained in RPMI 1640 medium containing 10% heat-inactivated FBS, 1 mM sodium pyruvate and 0.05 mM mercaptoethanol. THP-1 cells were stimulated for differentiation by PMA exposure (40 pmol/10^6^ cells) for 3 days. HL60 cells (North Nano Cell Bank, Shenzhen, China) were maintained in RPMI 1640. L1210 cells (North Nano Cell Bank) were maintained in DMEM (high glucose). 4T1 cells (North Nano Cell Bank) were maintained in RPMI 1640. All cell lines were tested for mycoplasma contamination and authenticated using the short tandem repeat method.

WT or *Tlr2* KO C57BL/6J mice (Jackson Laboratory, Bar Harbor, ME, USA, #004650) were pretreated with 1 mL 3% brewer thioglycollate medium for 4 days, i.p. and sacrificed using diethyl ether anesthesia. Cold PBS was injected intraperitoneally and peritoneal fluid was collected. Murine peritoneal macrophages (PMs) were harvested by centrifugation at 800 × *g* for 5 min and maintained in RPMI 1640.

Murine BMDMs were differentiated by M-CSF exposure for 7 days from BMs obtained from both femurs of C57BL/6J mice. BMDMs were maintained in DMEM/F-12.

All cells were maintained in medium containing 10% FBS and cultured in incubators with controlled temperature of 37 °C, 5% CO_2_, and 95% humidity.

### MEFs cultures and stable mutated-estrogen receptor (MER)-CreMER expression

MEFs cultures were established from *Gpx4*^flox/flox^ C57BL/6J mice (Jackson Laboratory, #027964) embryos at E12.5 as described previously [37]. MEFs were maintained in DMEM (high glucose).

*Gpx4*^flox/flox^ MEFs stably expressing Cre recombinase were established according to the procedure described previously [19, 38]. In brief, *Gpx4*^lox/flox^ MEFs were transfected by electroporation with plasmid pPBCAG-MERCreMER (Addgene, Watertown, MA, USA, #124183) or PBCAG-eGFP (Addgene, #40973) and puromycin acetyltransferase. *Gpx4* gene KO were induced with 4-hydroxytamoxifen through initiating the MERCreMER-mediated excision of floxed *Gpx4* alleles.

### Animal care

All C57BL/6J mice of WT, *Tlr2* KO, and *Gpx4*^flox/flox^ (Jackson Laboratory, Stock No.: 004650) were housed in-group in cages at a mean constant temperature (23 ± 2 °C), humidity (55 ± 5%), and illumination (12 h light-dark cycle), and free access to standard pellet chow and water. Mice were adapted to the facilities for 1 week before experiments.

### Assessment of phagocytosis in vitro

Cells were dispensed in dishes at a density of 5 × 10^5^ cells. HL60/L1210 cells were treated with RSL3 or STS for 6 h and incubated with CMFDA (1 μM) for 30 min. PMA-activated THP-1 cells were labeled with CD11b antibody (0.5 μg/mL) for 30 min (flow cytometry) or CellTracker Red CMTPX (1 μM) for 30 min (confocal microscopy). PMs and BMDMs were labeled with F4/80 (0.5 μg/mL) for 30 min. HL60/L1210 cells were collected and added to adherent macrophages (10:1) for 1.5-h incubation. Afterward, the suspended/unengulfed HL60/L1210 cells were removed and the macrophages were collected for analysis by a BD FACSCanto II flow cytometer (Becton, Dickinson and Company, Franklin Lakes, NJ, USA) equipped with FlowJo X 10.0.7 R2 (Becton Dickinson, Ashland, OR, USA), or imaged by a ZEISS LSM 800 confocal laser scanning microscope (Carl Zeiss AG, Oberkochen, Germany). The excitation and emission wavelengths of CMFDA are 492 and 517 nm, respectively. The excitation and emission wavelengths of CMTPX are 577 and 602 nm, respectively.

### Assessment of cell viability

Cells were dispensed in a 96-well plate at a density of 1 × 10^5^ cells per well. After 24-h incubation, cells were treated with the tested reagents for the indicated periods of time and stained with Cell Counting Kit-8. Optical density was measured using an ELISA reader (Thermo Fisher Scientific, Waltham, MA, USA).

### Quantitative polymerase chain reaction

HL60 were treated with RSL3 or STS for 24 h. Cells were collected and the RNA samples were prepared by extracting with Trizol on ice for 5 min, and the supernatants were collected after centrifugation at 13,000 × *g* for 15 min. Real-time polymerase chain reaction was performed following reverse transcription and analyzed using the CFX Connect Real-Time System (Bio-Rad Laboratories, Hercules, CA, USA). Band intensities were quantified by Quantity One (Bio-Rad Laboratories) and expressed as the ratio to 18S. The primer sequences are listed in Supplementary Methods.

### Western blotting

HL60 were treated with RSL3 or STS for 24 h. Cells were collected and resuspended in RIPA lysis buffer on ice for 5 min, and the supernatants were collected after centrifugation at 13,000 × *g* for 15 min. Protein samples were quantified using Pierce BCA protein assay kit. Protein lysates (30 μg) were separated in 8–15% gradient SDS-PAGE gels and blotted onto Immobilon-P PVDF membrane (MilliporeSigma, Burlington, MA, USA). Protein expression was detected using polyclonal antibody and visualized using mouse anti-rabbit IgG followed by HRP-conjugated anti-mouse IgG and Pierce ECL western blotting substrate (Thermo Fisher Scientific). Immunoblots were imaged on a Tanon 5200 Chemiluminescence Image Analysis System (Tanon Science & Technology, Shanghai, China).

### Plasma membrane isolation and validation

Plasma membranes of HL60 cells were isolated by Minute Plasma Membrane Protein Isolation Kit (Invent Biotechnologies, Blymouth, MA, USA) according to manufacturers’ instructions. The obtained plasma membrane preparations were further used for PLs by LC–MS/MS, after the purity was identified by western blot analysis using antibodies against markers of various biofilms, including plasma membrane (cadherin [39]), cytoplasm (*β*-actin), endoplasmic reticulum (calnexin [40]), mitochondria (VDAC [41]), Golgi apparatus (GM130 [42]), as well as the nucleus marker (histone H3 [43]).

### PI staining

Cells were dispensed in (confocal) dishes at a density of 5 × 10^5^ cells. After treatment, cells were incubated with 0.05 mg/mL propidium iodide (PI) (Sigma) for 30 min at 4 °C and then collected for analysis by a BD FACSCanto II flow cytometer, or imaged by a ZEISS LSM 800 confocal laser scanning microscope. The excitation and emission wavelengths of PI are 536 and 617 nm, respectively.

### Assessment of lipid peroxidation

Cells were dispensed in (confocal) dishes at a density of 5 × 10^5^ cells. HL60 cells received RSL3 for 6 h or for various times as described in the “Results” section, and *Gpx4*^flox/flox^ MEFs expressing Cre received 4-OHT for 48 h. After treatment, cells were incubated with Liperfluo (10 μM) for 1 h and DiD perchlorate (1 μM) for 30 min. Then, the cells were collected for analysis in a BD FACSCanto II flow cytometer, or imaged using a ZEISS LSM 800 confocal laser scanning microscope. The excitation and emission wavelengths of the oxidized Liperfluo are 524 and 535 nm, respectively. The excitation and emission wavelengths of DiD perchlorate are 644 and 663 nm, respectively.

### Assessment of PS externalization by flow cytometry

Cells were dispensed in dishes at a density of 5 × 10^5^ cells and treated with RSL3 (1 μM) or STS (0.25 μM) for 3, 6, 12, and 24 h. Then, the cells were incubated with annexin V-FITC and PI at 4 °C for 30 min, according to the procedure described in Beyotime Apoptosis Detection Kit (Shanghai, China). The cells were washed and analyzed by a BD FACSCanto II flow cytometer equipped with FlowJo X 10.0.7 R2.

### Preparation of lipids for LC–MS analysis

PLs or fatty acids were extracted with the reported Folch procedure [44] and were further analyzed by LC–MS using normal-phase (silica) or reverse-phase (C18) chromatography system coupled with Q-Exactive Hybrid Quadrupole-Orbitrap mass spectrometer (Thermo Fisher Scientific) or SCIEX Triple Quad 3500 system (Danaher Corporation).

### LC–MS/MS-based phospholipidomics analysis of plasma membrane isolated from HL60 cells treated with RSL3

PLs were separated by a Dionex UltiMate 3000 DGLC standard system (Thermo Fisher Scientific) at a flow rate of 0.2 mL/min on normal-phase column (Phenomenex Luna Silica, 3 μM, 150 × 2.0 mm, Danaher Corporation, Washington DC, USA). The column temperature was maintained at 35 °C. The mobile phase consisted of 10 mM ammonium formate in propanol/hexane/water (285:215:5, v/v/v, solvent A) and 10 mM ammonium formate in propanol/hexane/water (285:215:40, v/v/v, solvent B). The linear gradient conditions were as following: 0 min, 10% B; 20 min, 32% B; 30 min, 70% B; 32 min, 100% B; 58 min, 100% B; 60 min, 10% B; and 75 min, 10% B. The injection volume was 2 μL.

MS/MS analysis of PLs was performed on a Q-Exactive Hybrid Quadrupole-Orbitrap mass spectrometer (Thermo Fisher Scientific). Analysis was performed in full MS negative mode at resolution setting of 70,000 and data-dependent-MS/MS mode at resolution setting of 17,500. For MS, the scan range was 400–1800 *m*/*z* and the maximum ion injection time was 600 ms using 1 microscan per MS scan. For MS/MS, high energy collision induced dissociation (HCD) analysis was performed with the collision energy set to 24 eV and the maximum ion injection time of 200 ms. The inclusion list included all species of PLs and their oxidized/deuterated products. An isolation window of 1.0 Da was set for the MS and MS/MS scans. Capillary spray voltage was 3.0 kV, and capillary temperature was 320 °C. The S-lens Rf level was 60.

### SAPE-OOH quantification in *Gpx4* KO MEFs and tumor-bearing BALB/c mice by LC–MS/MS

LC–MS/MS was performed using Dionex UltiMate 3000 UHPLC standard system (Thermo Fisher Scientific) coupled with an SCIEX Triple Quad 3500 system (Danaher Corporation). Separation of the oxPLs was achieved by loading samples onto a reverse column (ACQUITY HSS T3, 1.8 μM, 50 × 2.0 mm, Waters Corporation, Milford, MA, USA). Elution of PLs was achieved using a binary gradient with solvent A (69% water; 31% methanol; 10 mM ammonium formate) and solvent B (50% methanol; 50% isopropanol; 10 mM ammonium formate) as the mobile phases with the column temperature maintained at 35 °C. The flow rate was 0.2 mL/min following the injection volume of 2 μL. The linear gradient conditions were as follows: 0 min, 80% B; 6 min, 80% B; 8 min, 100% B; 12 min, 100% B; 13 min, 80% B; and 25 min, 80% B. Ion pairs of SAPE-OOH selected for the MRM mode were *m*/*z* 798.6–283.3.

### AA-OOH, lysoPE, and SAPE-OOH quantification of plasma membrane of HL60 cells

AA-OOH was separated by a Vanquish UHPLC standard system (Thermo Fisher Scientific) at a flow rate of 0.25 mL/min on reverse-phase column (ACQUITY BEH C18, 1.8 μM, 100 × 2.0 mm, Waters Corporation, Milford, MA, USA). The column temperature was maintained at 35 °C. The mobile phase consisted of water containing 0.1% formic acid (solvent A) and acetonitrile (solvent B). The linear gradient conditions were as following: 0 min, 65% B; 4.5 min, 65% B; 8 min, 95% B; 13 min, 95% B; 13.5 min, 65% B; and 18 min, 65% B. The flow rate was 0.3 mL/min following the injection volume of 2 μL.

MS/MS analysis of AA-OOH was performed on a Q-Exactive Hybrid Quadrupole-Orbitrap mass spectrometer (Thermo Fisher Scientific). Full mass scan was performed in negative mode at resolution setting of 35,000 and data-dependent-MS/MS mode at resolution setting of 17,500. For MS, the scan range was 150–800 *m*/*z* with a maximum ion injection time of 200 ms using 1 microscan per MS scan. For MS/MS, HCD analysis was performed with the collision energy set to 20, 28, and 35 eV and the maximum ion injection time of 100 ms. An isolation window of 1.0 Da was set for the MS and MS/MS scans. Capillary spray voltage was 3.0 kV, and capillary temperature was 320 °C. The S-lens Rf level was 60.

LysoPE and SAPE-OOH were separated by a Dionex UltiMate 3000 DGLC system (Thermo Fisher Scientific) at a flow rate of 0.35 mL/min on normal-phase column (Phenomenex Luna Silica, 3 μM, 150 × 2.0 mm, Danaher Corporation, Washington DC, USA). The column temperature was set at 40 °C. The mobile phase consisted of 10 mM ammonium formate in propanol/hexane/water (285:215:5, v/v/v, solvent A) and 10 mM ammonium formate in propanol/hexane/water (285:215:40, v/v/v, solvent B). The linear gradient conditions were as follows: 0 min, 10% B; 5 min, 45% B; 6 min, 100% B; 13 min, 100% B; 13.5 min, 10% B; and 18 min, 10% B. The injection volume was 2 μL.

MS/MS analysis of lysoPE and SAPE-OOH is consistent with the phospholipidomics assay.

### Proteomics

HL60 received RSL3 for 6 h to promote ferroptosis. THP-1 cells were pretreated with PMA (40 pmol/10^6^ cells) for 3 days and incubated with ferroptotic cells for 1.5 h at 37 °C. Unengulfed HL60 cells were removed. Then, the THP-1 cells were treated with 50 μM biotin or SAPE-biotin in RPMI 1640 (without FBS) for 4 h, and then harvested and incubated with prewashed streptavidin beads (Thermo Fisher Scientific) overnight at 4 °C on a shaker. Proteins interacted with SAPE-biotin were eluted by Laemmli buffer containing 500 μL 6 M urea, 25 μL 200 mM DTT, and 25 μL 500 mM IAA in dark at room temperature for 30 min. The eluent was incubated with 150 μL 2M urea, 150 μL 1 mM CaCl_2_, and 1 μL trypsin (1 μg/μL) at 37 °C overnight. After that, the protein samples were purified by ODS C18 SPE column (Agilent) and analyzed by LC–MS/MS. Samples were then analyzed in a data-dependent acquisition mode by the LC−MS/MS, equipped with an EASY-nLC 1200 (Thermo Fisher Scientific) HPLC system and Orbitrap Fusion Lumos (Thermo Fisher Scientific) mass spectrometer. For LC separation, tryptic peptides were sequentially injected into an Acclaim PepMap 100 C18 column (100 μM × 2 cm, 5 μM, Thermo Fisher Scientific, P/N:164564) and an Acclaim PepMap 100 C18 column (50 μM × 15 cm, 2 μM, Thermo Fisher Scientific, P/N:164943).

### Molecular docking

The X-ray crystal structure of TLR2 (PDB ID: 2Z80) was downloaded from the Protein Data Bank (https://www.rcsb.org/). Molecular docking was carried on CDOCKER module of Accelrys Discovery Studio (version 3.5; Accelrys, San Diego, CA, USA). The protein and compounds were processed by CHARMM force field. Docking parameters were set according to the standard values. Docking modes and scores were analyzed after molecular docking completed.

### Pull-down assay for TLR2

HL60 received RSL3 for 6 h to induce ferroptosis. THP-1 cells were pretreated with PMA (40 pmol/10^6^ cells) for 3 days and incubated with ferroptotic cells for 1.5 h at 37 °C. Unengulfed HL60 cells were removed. Then, the THP-1 cells were treated with 50 μM biotin or SAPE-biotin in RPMI 1640 (without FBS) for 4 h, and then harvested and incubated with prewashed streptavidin beads (Thermo Fisher Scientific) overnight at 4 °C on a shaker. The beads were collected and heated to 100 °C for 10 min, and then loaded on SDS-PAGE.

For protein gel staining, gels were incubated with Coomassie Brilliant Blue staining solution for 6 h, and then washing five times with 1% phosphoric acid (H_3_PO_4_).

For western blotting, proteins were transferred from gel to Immobilon-P PVDF membrane (MilliporeSigma). Proteins expression were detected using TLR2 antibody and visualized using secondary antibody conjugated with HRP and Pierce ECL western blotting substrate (Thermo Fisher Scientific) as the substrate of HRP. The immunoblot was detected by Tanon 5200 Chemiluminescence Image Analysis System (Tanon Science & Technology).

### Lipid dot-blot assay

Lipids (2.7 nmol, including SAPE, SAPE-OOH, SAPS, and SAPC) were immobilized on Immobilon-P PVDF membrane (MilliporeSigma) and then incubated with 5.8 nM recombinant TLR2 protein (2616-TR, R&D Systems, Bio-Techne Corporation, Minneapolis, MN, USA) in PBS. Afterward, membranes were incubated with TLR2 antibody and visualized using secondary antibody conjugated with HRP and Pierce ECL western blotting substrate (Thermo Fisher Scientific) as the substrate of HRP. The immunoblot was detected by Tanon 5200 Chemiluminescence Image Analysis System (Tanon Science & Technology).

### Cellular thermal shift assay

THP-1 cells were pretreated with PMA (40 pmol/10^6^ cells) for 3 days. Cells were harvested and lysed by three cycles of flash-freeze–thawing using liquid nitrogen and room temperature water. The supernatants were collected after centrifugation at 13,000 × *g* for 15 min and were quantified using Pierce BCA protein assay kit. Cells supernatants were incubated with DMSO, SAPE (2.5 μM), and SAPE-OOH (2.5 μM) at 37 °C for 1 h. Ligand-binding-induced protein thermal stabilization was evaluated followed the melting curve analysis using 37–73 °C gradient for 3 min. After cooling down at room temperature for 3 min, the protein samples were analyzed by western blot analysis.

### *Tlr2* knockdown with siRNA

PMA (40 pmol/10^6^ cells) differentiated THP-1 cells were seeded overnight before transfection. siRNA and Lipofectamine™ 2000 were diluted in Opti-MEM media (Life Technologies, Gaithersburg, MD, USA) at a final concentration of 50 nM, mixed and incubated for 20 min to obtain the Opti-MEM-siRNA mixture. Cells were incubated with the mixture for 6 h and then cultured in PMA-containing (40 pmol/10^6^ cells) fresh full media for 42 h. The sequences of siRNA were listed in Supplementary Methods.

### Tumor-bearing BALB/c mice

#### Treatment

BALB/c mice (female, 8-week old, Guangdong Medical Laboratory Animal Center, Guangdong, China) were injected with 4T1 cells (5 × 10^5^ cells) in the mammary fat pad. Once the tumor sizes reached ~100 mm^3^, the mice were injected with saline (200 μL), RSL3 (100 mg/kg), or RSL3 (100 mg/kg) + rosiglitazone (30 mg/kg) intratumorally twice a week for 2 weeks according to the reported research [6].

#### Tumor weight/volume

During treatment period, the body weight of the 4T1-bearing mice was recorded every day, and tumor volume of each mouse was calculated according to the following equation: tumor volume (mm^3^) = [tumor length (mm)] × [tumor width (mm)]/2. The mice were euthanatized at the end of administration. The tumor tissues were weighted and fixed in 4% paraformaldehyde.

### Tumor tissue staining

#### TUNEL assay

Fixed tumor tissues were paraffin embedded, cut into 4 μM slices, and then processed using standard deparaffinization and rehydration techniques. The slices were stained by H&E, before performing the TUNEL assay as described in the In Situ Cell Death Detection Kit (Roche Applied Science, Penzberg, Upper Bavaria, Germany). The stained slices were imaged by a M8 Digital Microscope and Scanner (PreciPoint GmbH, Freising, Germany).

#### Immunohistochemistry

Fixed tumor tissues were paraffin embedded, cut into 4 μM slices, and then processed using standard deparaffinization and rehydration techniques. The slices were stained by H&E, followed by labeling with GPX4, 4-HNE, or F4/80 antibodies. Primary antibodies were visualized using HRP-conjugated secondary antibodies and DAB as the substrate. Slices were imaged using a M8 Digital Microscope and Scanner (PreciPoint GmbH).

### MDA assay

The level of MDA was determined by the TBARS assay according to the protocol provided by the manufacturer (Beyotime Biotechnology, Shanghai, China). After treatment, tumor tissues collected and lysed with sonication. The homogenate of 10% was prepared by a homogenizer. Then, the samples were centrifuged at 4 °C, and the supernatants were collected for detection of MDA content using an ELISA plate reader. The concentration of MDA was calculated according to a previously prepared standard curve (MDA concentration range from 1 to 50 µM).

### Assessment of phagocytosis in vivo

WT or *Tlr2* KO C57BL/6J (female, 8-week old) mice were injected i.p. with 1 mL 3% brewer thioglycollate medium to raise macrophage output. After 4 days, 5 × 10^6^ CMFDA-labeled L1210 cells previously treated with RSL3 (5 μM, 6 h) or SAPE-OOH (10 μM, 6 h) were injected into the mouse peritoneum. Total peritoneal cells were harvested 24 h later using cold PBS containing 2% fetal bovine serum and 1 mM EDTA, and stained with F4/80 (0.5 μg/mL) for 30 min to label PMs. Afterward, the cells were collected to assess in vivo phagocytic capacity by a BD FACSCanto II flow cytometer.

### Statistical analysis

The data were analyzed by IBM SPSS Statistics 25.0 (SPSS Inc., Chicago, IL, USA). The variance was similar between the groups that are being statistically compared. All data were expressed as means ± SEM of independent experiments. *P* values were determined by independent samples *t*-test, one-way ANOVA, with LSD post hoc and two-way repeated measures. *P* < 0.05 is considered statistically significant.

## Supplementary information

Supplementary figures

Supplementary methods
