# Supplementary material for: Oxygenated phosphatidylethanolamine navigates phagocytosis of ferroptotic cells by interacting with TLR2
Source: Cell Death Differ. 2021 Jan 11;28(6):1971–89. doi: 10.1038/s41418-020-00719-2 (PMC8185102; doi:10.1038/s41418-020-00719-2)
Supplement: Supplementary file 1 — Supplementary figures [file 41418_2020_719_MOESM1_ESM.docx]

**Oxygenated phosphatidylethanolamine navigates phagocytosis of ferroptotic cells by interacting with TLR2**

**Xiang Luo^1,2,*^ · Hai-Biao Gong^1,2,*^ · Hua-Ying Gao^1,2,*^ · Yan-Ping Wu^1,2,3^ · Wan-Yang Sun^1,2^ ·** **Zheng-Qiu Li^2^ · Guan Wang^4^ · Bo Liu^4^ · Lei Liang^1,2^ · Hiroshi Kurihara^1,2^ · Wen-Jun Duan^1,2,^**^🖂^ **· Yi-Fang** **Li^1,2,^**^🖂^ **· Rong-Rong He^1,2,3,^**^🖂^

Running title: Lipid peroxidation evokes uptake of ferroptosis.

^🖂^Lead Correspondence: He RR (rongronghe@jnu.edu.cn)

^🖂^Co-Correspondence: Duan WJ (duanwj@jnu.edu.cn), Li YF (liyifang706@jnu.edu.cn)

*These authors contributed equally: Xiang Luo, Hai-Biao Gong, Hua-Ying Gao

^1^ Guangdong Engineering Research Center of Chinese Medicine & Disease Susceptibility, Jinan University, Guangzhou 510632, China.

^2^ International Cooperative Laboratory of Traditional Chinese Medicine Modernization and Innovative Drug Development of Chinese Ministry of Education (MOE), College of Pharmacy, Jinan University, Guangzhou 510632, China.

^3^ Integrated Chinese and Western Medicine Department, School of Traditional Chinese Medicine, Jinan University, Guangzhou 510632, China.

^4^ State Key Laboratory of Biotherapy and Cancer Center, West China Hospital, Sichuan University, and Collaborative Innovation Center for Biotherapy, Chengdu 610041, China.

**Supplementary Figures**


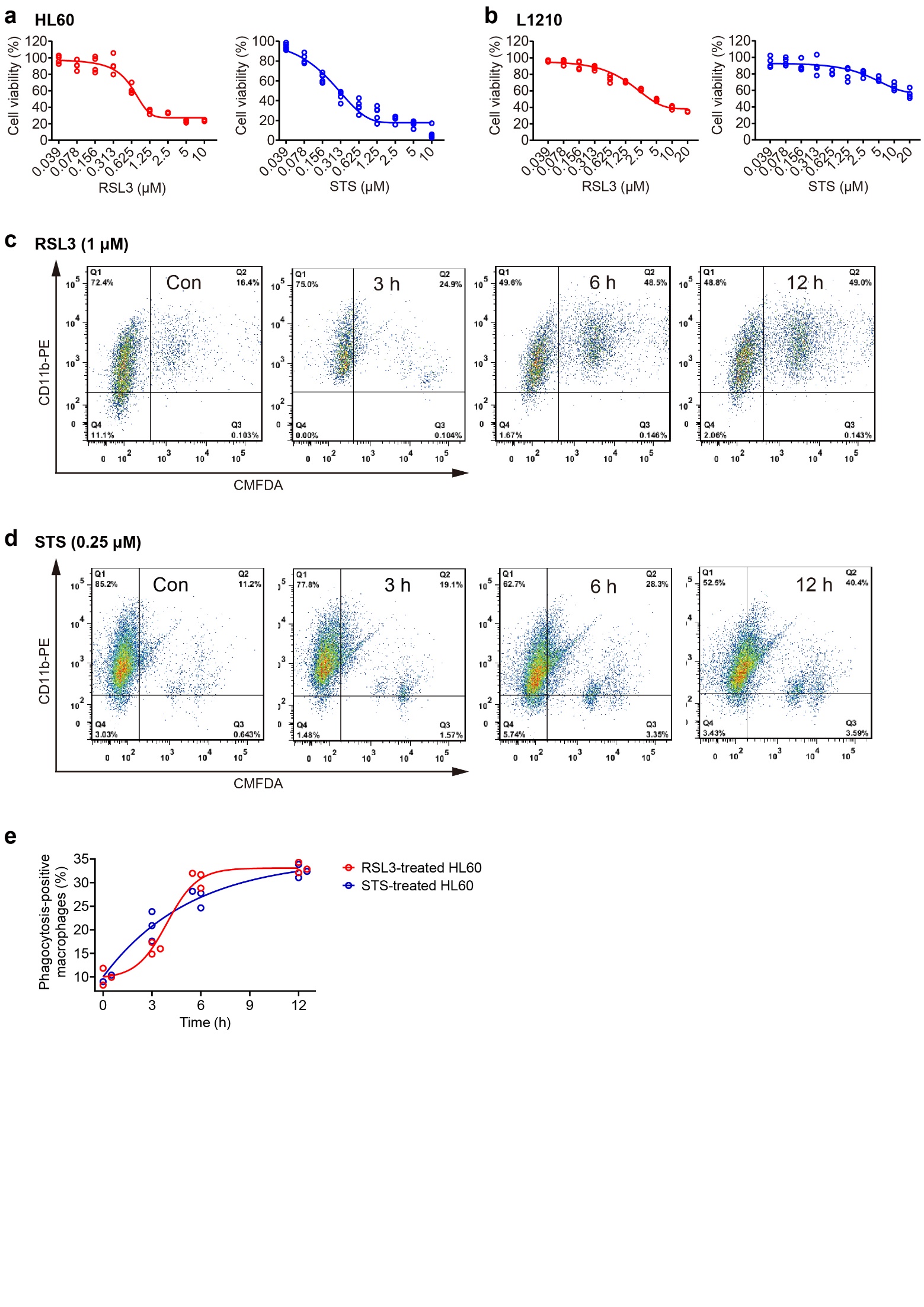


**Fig. S1. Ferroptotic and apoptotic cells are phagocytosed by macrophages.** HL60 (**a**) and L1210 (**b**) cells were treated with RSL3 and STS dose-dependently for 6 h, and cell viability was determined by CCK-8 assay. Data are mean ± SEM (*n* = 4-5 independent biologically cell cultures). CMFDA-labeled HL60 cells were treated with 1 μM RSL3 (**c**) and 0.25 μM STS (**d**) time-dependently before co-incubated with PMA-activated THP-1 cells (labelled with CD11b-PE antibody) for 1.5 h. (**e**) The phagocytosis was detected by flow cytometry and the data were quantitively determined. Data are mean ± SEM (*n* = 3 independent biologically cell cultures).


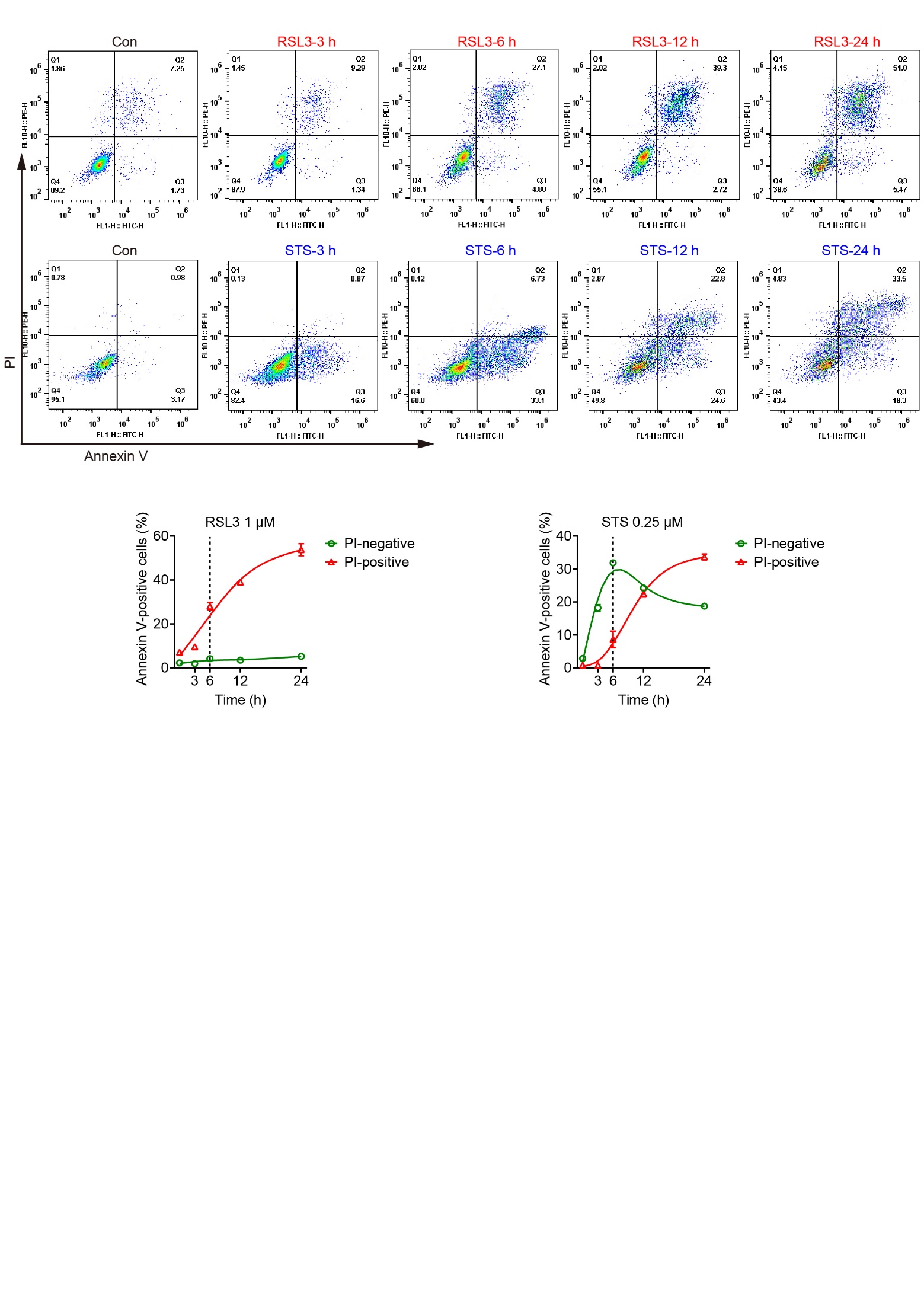


**Fig. S2. Cell surface expression of phosphatidylserine is unaffected in ferroptotic cells.** HL60 cells were treated with RSL3 (1 μM) or STS (0.25 μM) for different hours and then stained with Annexin V and PI. The positivity was measured by flow cytometry. Green: Annexin V^+^/PI^-^. Red: Annexin V^+^/PI^+^. Data are mean ± SEM (*n* = 3 independent biologically cell cultures).


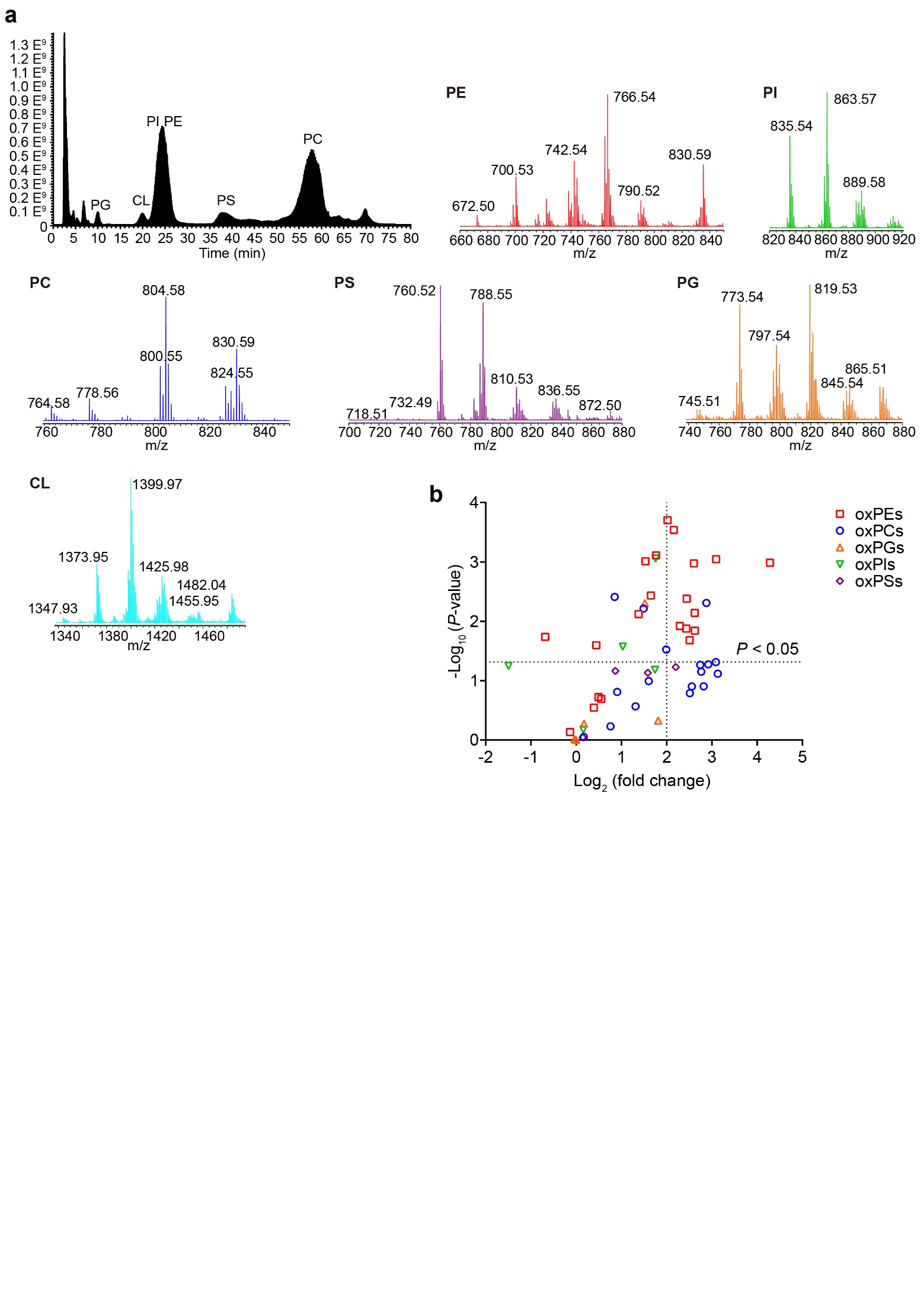


**Fig. S3. LC-MS/MS is utilized for determination of phospholipids.** (**a**) Representative normal phase LC-MS/MS chromatogram and mass spectra for 6 major classes of phospholipids in HL60 cells. PE, phosphatidylethanolamines. PC, phosphatidylcholine. PI, phosphatidylinositol. PS, phosphatidylserine. PG, phosphatidylglycerol. CL, cardiolipin. (**b**) Volcano plots of changes in the levels of oxygenated phospholipids (log_2_ (fold change), X-axis) *vs* significance (-log_10_ (*P*-value), Y-axis, by *t*-test) of RSL3 (1 μM, 6 h)-treated group and untreated group. ox, oxygenated. Each dot represents a class of phospholipids. Data are mean ± SEM (*n* = 3 independent biologically cell cultures).


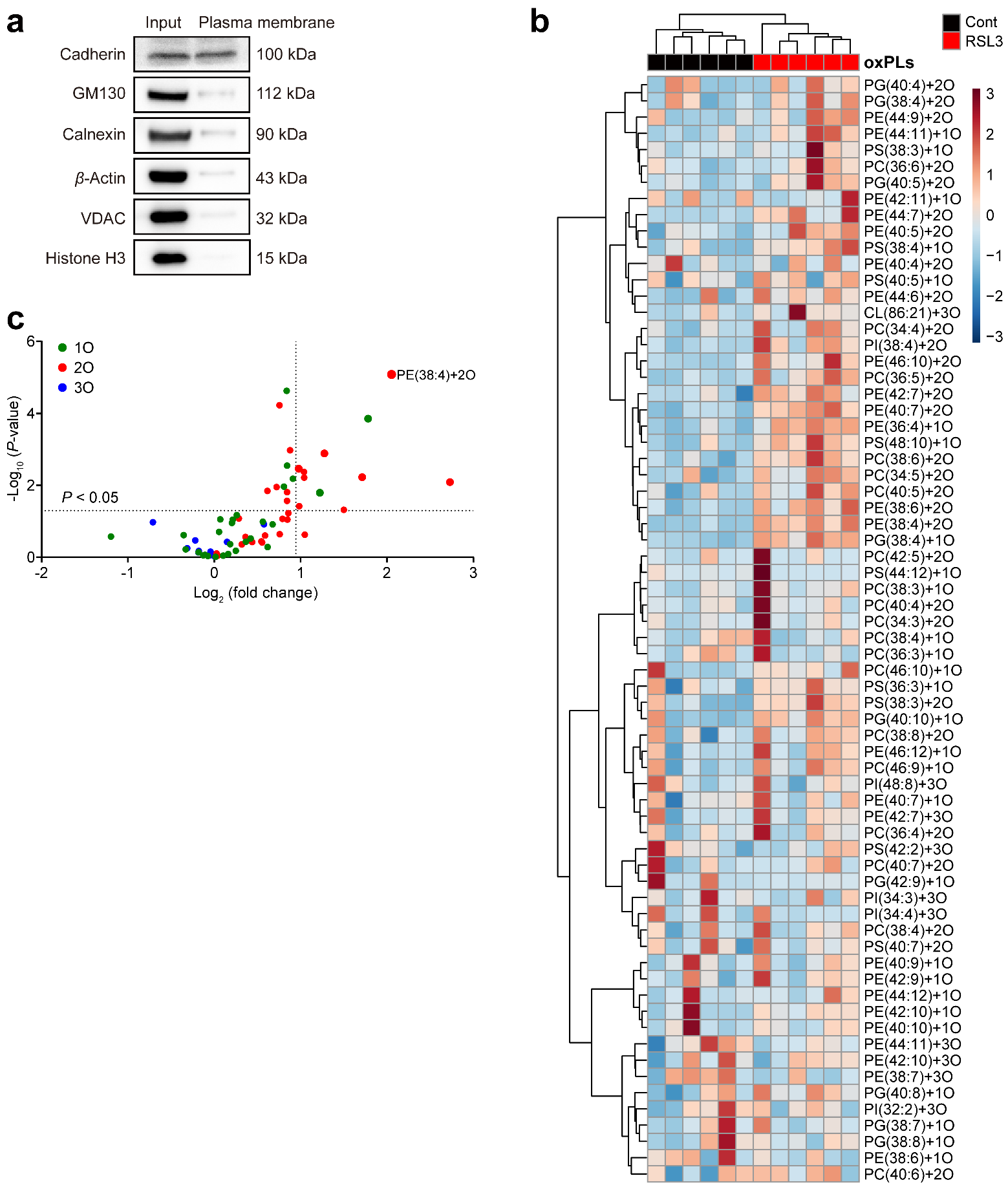


**Fig. S4. LC-MS/MS-based phospholipidomics were performed upon plasma membrane.** (**a**) The plasma membrane of HL60 cells was isolated and identified with multiple biofilm markers. (**b**) Oxygenated phospholipids were assessed by LC-MS/MS and the repertoires were displayed by heatmap. Data are mean ± SEM (*n* = 6 independent biologically cell cultures). Cont, control. RSL3, 1 μM, 6 h. (**c**) Obtained data were displayed by volcano plots showing the changes in the levels of oxygenated phospholipids (log_2_ (fold change), X-axis) *vs* significance (-log_10_ (*P*-value), Y-axis, by *t* test). 1O, 2O and 3O represents singly, doubly, and triply oxygenated PLs, respectively. Data are mean ± SEM (*n* = 6 independent biologically cell cultures).


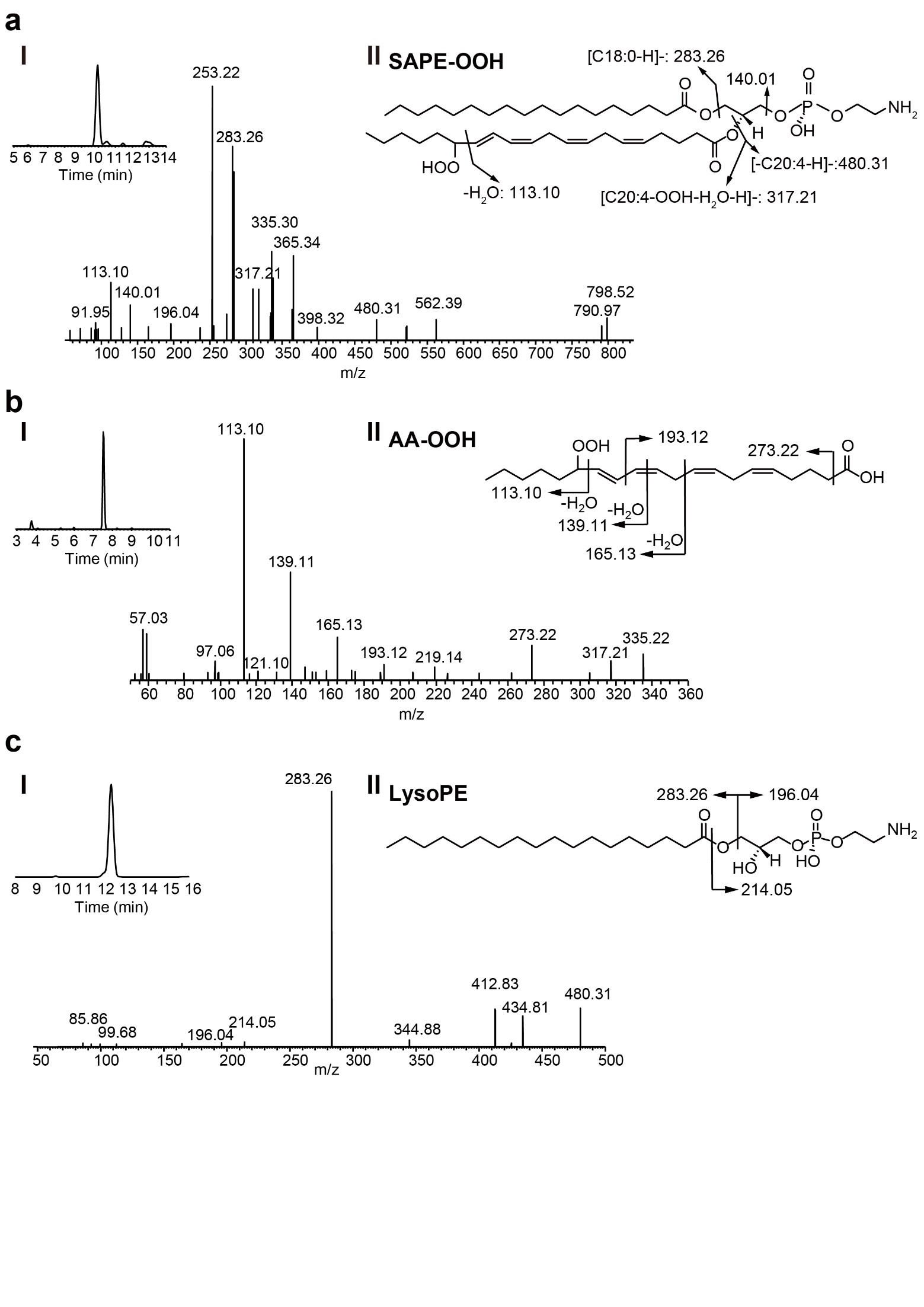


**Fig. S5**. **Composite MS/MS spectrum of SAPE-OOH (a, 1-stearoyl-2-15-HpETE-*sn*-glycero-3-phosphatidylethanolamine), AA-OOH (b) and lysoPE (c, 1-stearoyl-2-OH-*sn*-glycero-3-phosphatidylethanolamine).** (**I**) showed the extraction ion currents. (**II**) showed the structural formulas and fragments formed during MS/MS analysis.


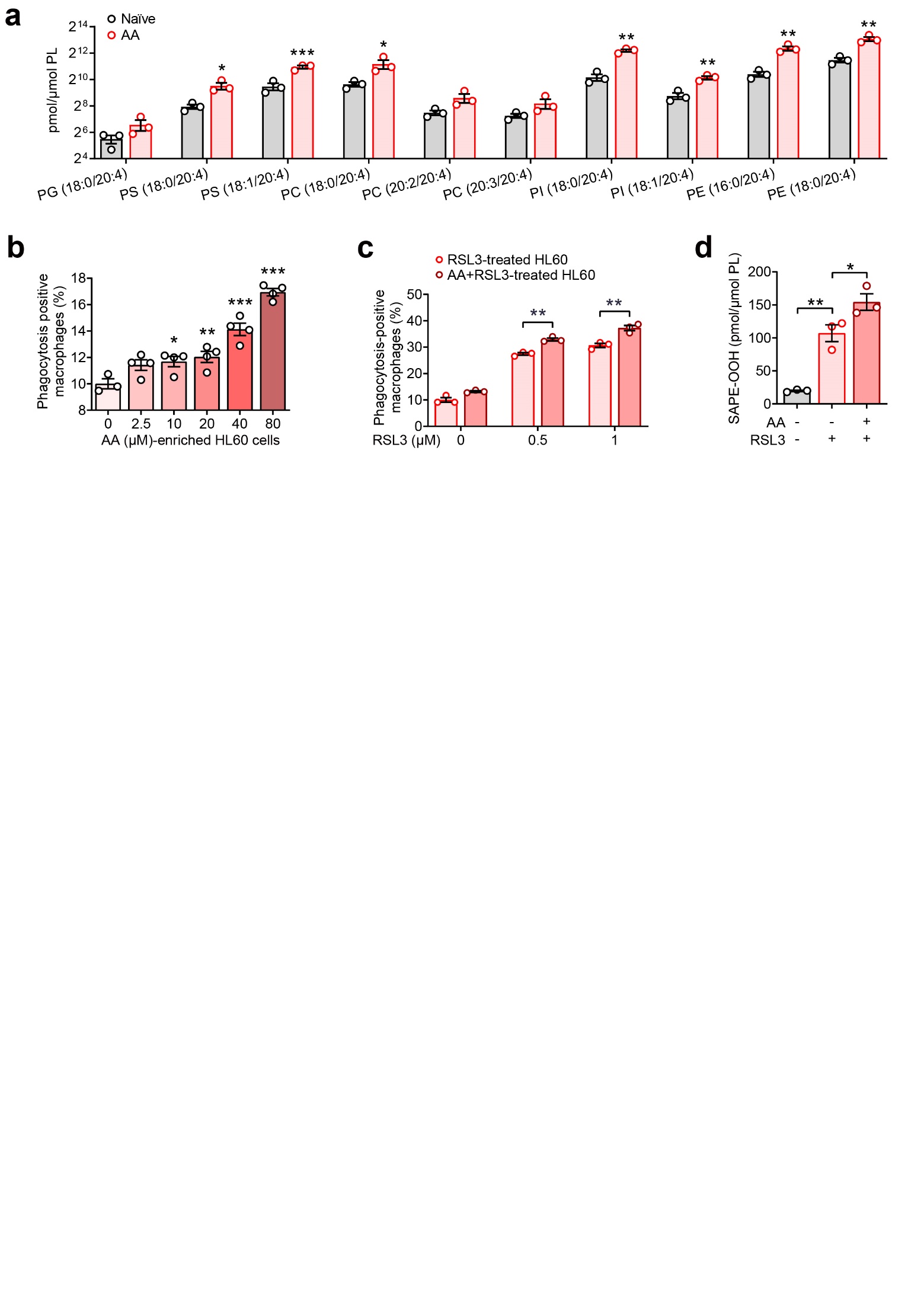


**Fig. S6. The relevance of esterified oxygenated PUFA to the ferroptotic mechanism.** (**a**) AA (10 μM, 16 h) contributes to enrichment of unsaturated fatty acids in all phospholipid classes (PLs) in HL60 cells. Data are mean ± SEM (*n* = 3 independent biologically cell cultures). (**b**) Comparison of the effect of dose-dependent enrichment of AA in HL60 cells on phagocytosis by PMA-activated THP-1 cells. Data are mean ± SEM (*n* = 3-4 independent biologically cell cultures). (**c**) Phagocytosis of HL60 cells treated with supplementary AA and dose-dependent RSL3 (6 h). Data are mean ± SEM (*n* = 3 independent biologically cell cultures). (**d**) HL60 cells were supplemented with exogenous arachidonic acid (AA, 10 μM) for 16 h followed by RSL3 treatment, and then the content of SAPE-OOH was determined by LC-MS/MS. Data are mean ± SEM (*n* = 3 independent biologically cell cultures). ^*^*P* < 0.05, ^**^*P* < 0.01 and ^***^*P* < 0.001, by 1-way ANOVA with LSD post-*hoc* test (for **a**, independent-samples *t*-test).


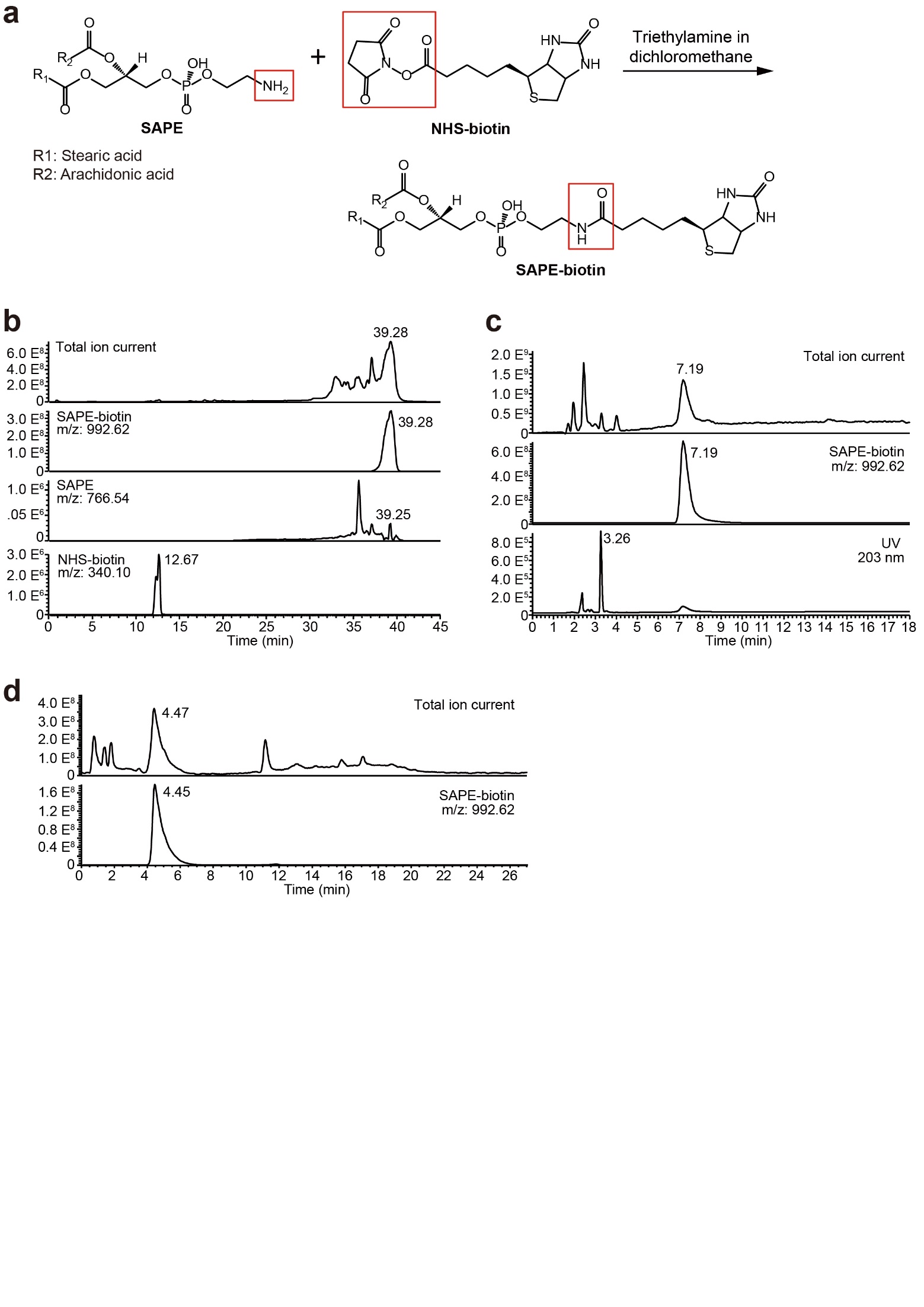


**Fig. S7. Preparation of SAPE-biotin.** (**a**) Synthetic scheme of SAPE-biotin. (**b**) Verification of SAPE-biotin by LC-MS/MS. (**c**) Purification of SAPE-biotin by HPLC. (**d**) Purity test of SAPE-biotin by LC-MS/MS.


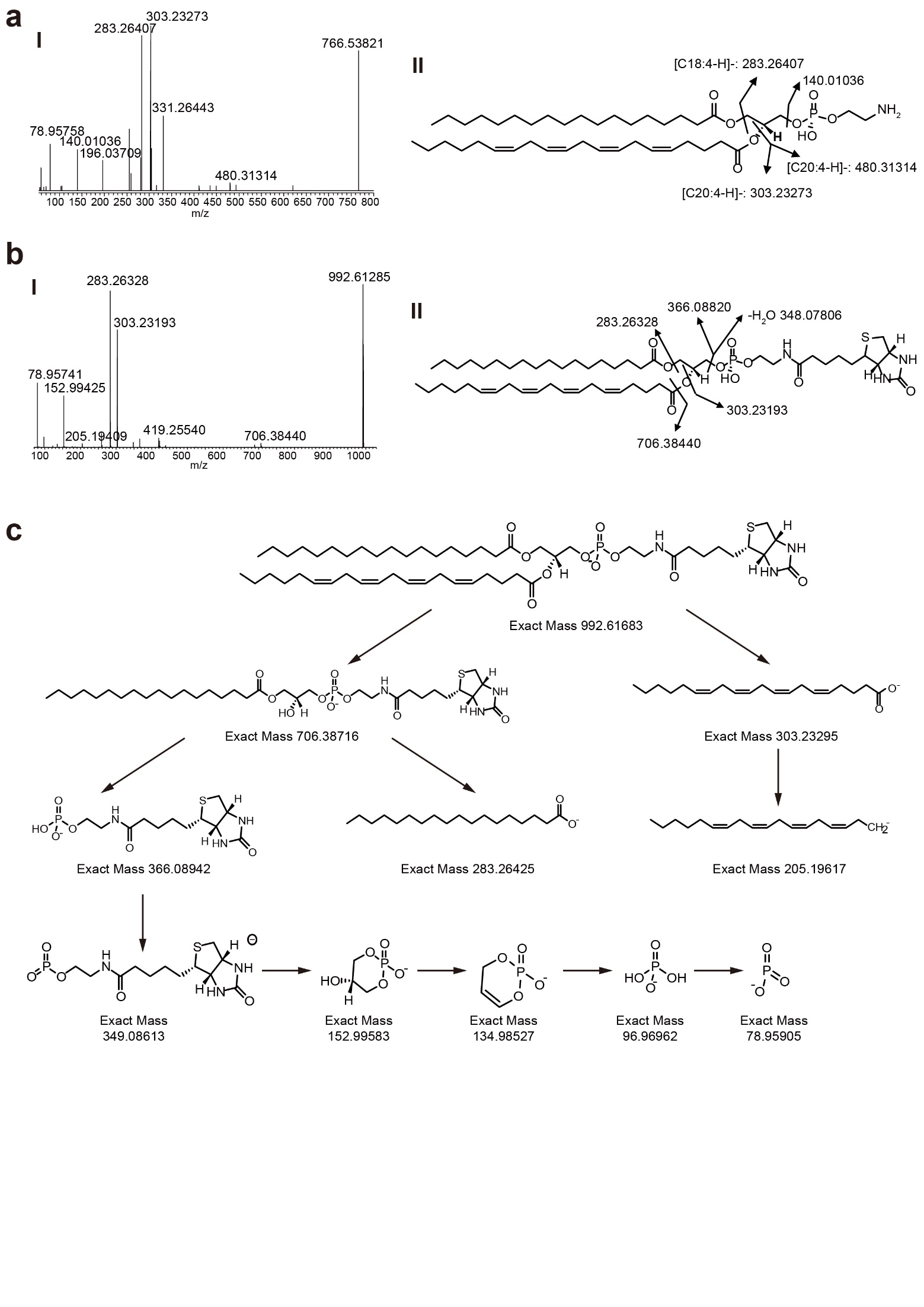


**Fig. S8**. **Identification of SAPE-biotin.** (**a**) MS/MS analysis of SAPE. (**b**) MS/MS analysis of SAPE-biotin. (**I**) showed the MS/MS spectrum. (**II**) showed the structural formulas and fragments formed during MS/MS analysis. (**c**) Fragmentation pathways of SAPE-biotin.


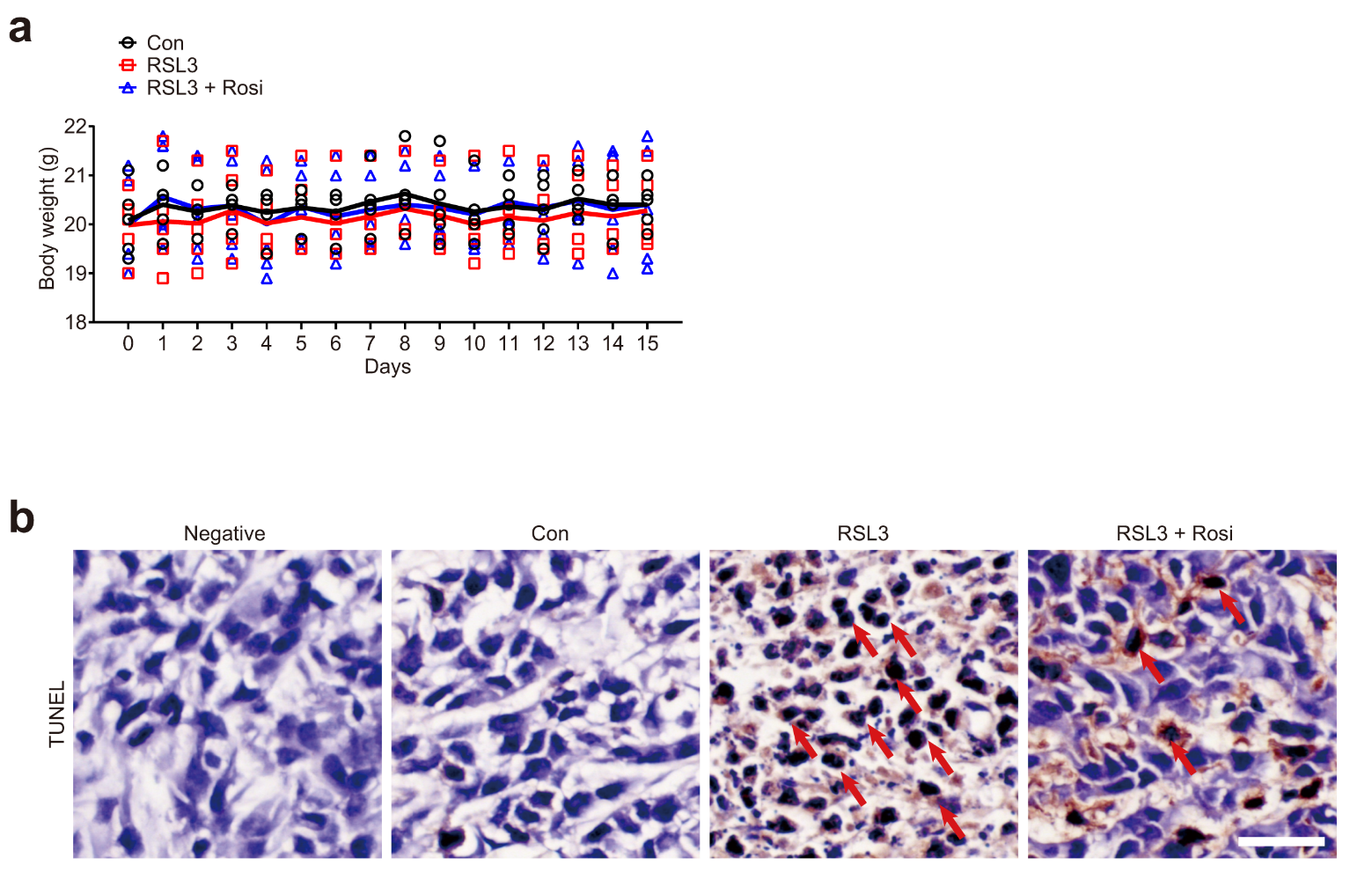


**Fig. S9**. **Data of mammary tumor-bearing mice.** (**a**) There was no difference in body weight between the three groups of mice. Data are mean ± SEM (*n* = 5 independent biologically animals). (**b**) TUNEL assay was carried out to evaluate the death ratio of tumor cells. Scale = 25 μm. Red arrows: TUNEL-positive cells.
