# Supplementary material for: Oxygenated phosphatidylethanolamine navigates phagocytosis of ferroptotic cells by interacting with TLR2
Source: Cell Death Differ. 2021 Jan 11;28(6):1971–89. doi: 10.1038/s41418-020-00719-2 (PMC8185102; doi:10.1038/s41418-020-00719-2)
Supplement: Supplementary file 2 — Supplementary methods [file 41418_2020_719_MOESM2_ESM.docx]

**cOxygenated phosphatidylethanolamine navigates phagocytosis of ferroptotic cells by interacting with TLR2**

**Xiang Luo^1,2,*^ · Hai-Biao Gong^1,2,*^ · Hua-Ying Gao^1,2,*^ · Yan-Ping Wu^1,2,3^ · Wan-Yang Sun^1,2^ ·** **Zheng-Qiu Li^2^ · Guan Wang^4^ · Bo Liu^4^ · Lei Liang^1,2^ · Hiroshi Kurihara^1,2^ · Wen-Jun Duan^1,2,^**^🖂^ **· Yi-Fang** **Li^1,2,^**^🖂^ **· Rong-Rong He^1,2,3,^**^🖂^

Running title: Lipid peroxidation evokes uptake of ferroptosis.

^🖂^Lead Correspondence: He RR (rongronghe@jnu.edu.cn)

^🖂^Co-Correspondence: Duan WJ (duanwj@jnu.edu.cn), Li YF (liyifang706@jnu.edu.cn)

*These authors contributed equally: Xiang Luo, Hai-Biao Gong, Hua-Ying Gao

^1^ Guangdong Engineering Research Center of Chinese Medicine & Disease Susceptibility, Jinan University, Guangzhou 510632, China.

^2^ International Cooperative Laboratory of Traditional Chinese Medicine Modernization and Innovative Drug Development of Chinese Ministry of Education (MOE), College of Pharmacy, Jinan University, Guangzhou 510632, China.

^3^ Integrated Chinese and Western Medicine Department, School of Traditional Chinese Medicine, Jinan University, Guangzhou 510632, China.

^4^ State Key Laboratory of Biotherapy and Cancer Center, West China Hospital, Sichuan University, and Collaborative Innovation Center for Biotherapy, Chengdu 610041, China.

**Supplementary Methods**

**List of resources**

| **Category** | **Company** | **Product No.** | |
| --- | --- | --- | --- |
| ***Chemicals*** |  |  | |
| RSL3 | Selleckchem | S8155 | |
| Staurosporine (STS) | Selleckchem | S1421 | |
| Ferrostatin-1 (Fer-1) | Selleckchem | S7243 | |
| Deferoxamine mesylate (DFO) | Selleckchem | S5742 | |
| Vitamin E (Vit. E) | Selleckchem | S4686 | |
| zVAD-FMK (zVAD) | Selleckchem | S7023 | |
| Dithiothreitol (DTT) | MCE | HY-15917 | |
| Indole-3-acetic acid (IAA) | Bioruler | RH31900 | |
| Urea | Aladdin | U111898 | |
| 4-hydroxytamoxifen (4-OHT) | MCE | HY-16950 | |
| Arachidonic acid (AA) | TargetMol | T4129 | |
| Bovine Serum Albumin (BSA) | Sigma-Aldrich | A1933 | |
| 3,3' Diaminobenzidine Tetrahydrochloride (DAB) | HYCX | HY-15912 | |
| NHS-Biotin | ApexBio | A8002 | |
| CU-CPT22 | Selleckchem | S8677 | |
| Rosiglitazone (Rosi) | Aladdin | R128083 | |
| 1-stearoyl-2-arachidonoyl-sn-glycero-3-phosphoethanolamine (SAPE) | Avanti | 850804 | |
| ***Recombinant Proteins and fluorogenic dyes*** | | |  |
| PMA | Sigma-Aldrich | P1585 | |
| M-CSF | ThermoFisher | MA523708 | |
| Recombinant human TLR2 | R&D Systems | 2616-TR-050 | |
| lipoprotein-associated phospholipase A2 (Lp-PLA_2_) | R&D Systems | 5106-PL-010 | |
| Liperfluo | Dojindo | L248 | |
| BES-H_2_O_2_-Ac | Wako | 028-17811 | |
| DiD (2-[5-(1,3-Dihydro-3,3-dimethyl-1-octadecyl-2H-indol-2-ylidene)-1,3-pentadien-1-yl]-3,3-dimethyl-1-octadecyl-3H-indolium perchlorate) | Meilunbio | MB6190 | |
| MitoTracker Red CMXRos | Invitrogen | M7512 | |
| CellTracker Green CMFDA | Invitrogen | C2925 | |
| CellTracker Red CMTPX | Invitrogen | C34552 | |
| FITC anti-mouse F4/80 | Biolegend | 123116 | |
| PE anti-human CD11b | Biolegend | 301306 | |
| DAPI | Beyotime | C1002 | |
| ***Critical Commercial kits*** | | |  |
| Cell counting kit-8 | Dojindo | CK04 | |
| MDA assay kit | Beyotime | S0131 | |
| Reverse transcription kit | TransGen | AT311-03 | |
| Apoptosis detection kit | Beyotime | C1065L | |
| Hematoxylin and eosin (H&E) staining kit | Beyotime | C0105 | |
| *In situ* cell death detection kit  Minute plasma membrane protein isolation kit | Roche  Invent | 1168479590  SM-005 | |
| ***Antibodies*** | | |  |
| Rabbit anti-GPX4 | Abcam | ab125066 | |
| Rabbit anti-4-HNE | Abcam | ab46545 | |
| Rabbit anti-CD47 | Abcam | ab108415 | |
| Rabbit anti-F4/80 | Abcam | ab6640 | |
| Rabbit anti-CALR | CST | 12238S | |
| Goat anti-TLR2  Rabbit anti-Cadherin  Rabbit anti-Calnexin  Rabbit anti-VDAC  Rabbit anti-GM130  Rabbit anti-Histone H3 | R&D Systems  Abcam  Abcam  CST  Abcam  Abcam | AF2616  ab76011  ab22595  4661s  ab52649  ab8898 | |
| Mouse anti-*β*-Actin | FuDe | FD0060 | |
| Mouse anti-GAPDH | FuDe | FD0063 | |
| Goat anti mouse-HRP | FuDe | FDM007 | |
| Goat anti rabbit-HRP | FuDe | FDR007 | |
| Alexa Fluor 555 goat anti-rabbit IgG (H+L) | Invitrogen | A21428 | |
| ***Others*** | | |  |
| KA1153_pPBCAG-MerCreMer-IN | Addgene | 124183 | |
| PBCAG-eGFP | Addgene | 40973 | |
| RIPA lysis buffer | Beyotime | P0013C | |
| Trizol | Invitrogen | 15596018 | |
| Fetal bovine serum (FBS) | PAN | P30-3302 | |

**List of primer sequences used in quantitative polymerase chain reaction**

| **Gene name** | **Primer sequence** |
| --- | --- |
| *HSP90AA1* | Forward primer, 5’-ATGGCAGCAAAGAAACAC-3’ |
|  | Reverse primer, 5’-GTATCATCAGCAGTAGGGTCA-3’ |
| *MFGE8* | Forward primer, 5’-CCTGCCACAACGGTGGTTTAT-3’ |
|  | Reverse primer, 5’-GCGATCTGTGAGTTGGCAATGT-3’ |
| *GAS6* | Forward primer, 5’-CATCAACAAGTATGGGTCTCCGTA-3’ |
|  | Reverse primer, 5’-TTGGGTCCCCTTCCTATCGC-3’ |
| *ICAM3* | Forward primer, 5’-CAATCTCAGCAACGTGACTGGCAA-3’ |
|  | Reverse primer, 5’-ACGGTGATGTTAGAGGAGCCTGTT-3’ |
| *C1QB* | Forward primer, 5’-GGCTTCCAGGGCTGGCTGGAG-3’ |
|  | Reverse primer, 5’-TCCCGATTCACCTTTGGGGCC-3’ |
| *CALR* | Forward primer, 5’-CATGTCTGTCTGGTCCAAACTATTA-3’ |
|  | Reverse primer, 5’-GCACGCTTTTATGCTCTGTCG-3’ |
| *VISR* | Forward primer, 5’-GTGAAGGTCCTGGAACGTGAG-3’ |
|  | Reverse primer, 5’-CGCCGTATTCCCTGTATGTCT-3’ |
| *18S* | Forward primer, 5’-CAGTAAGTGCGGGTCATAAGC-3’ |
|  | Reverse primer, 5’-CTCACTAAACCATCCAATCGG-3’ |

**List of siRNAs of *TLR2***

| **siRNA No.** | **Primer sequence** |
| --- | --- |
| si*TLR2*-1 | Forward primer, 5’-CCAAUGGAAUUAACACAAU-3’ |
|  | Reverse primer, 5’- AUUGUGUUAAUUCCAUUGG-3’ |
| si*TLR2*-2 | Forward primer, 5’-CCCUCUCUACAAACUUUAA-3’ |
|  | Reverse primer, 5’-UUAAAGUUUGUAGAGAGGG-3’ |
| si*TLR2*-3 | Forward primer, 5’-CCUCUUACCCAUGUUACUA-3’ |
|  | Reverse primer, 5’-UAGUAACAUGGGUAAGAGG-3’ |

**Synthesis of SAPE-biotin**

The synthesis was carried out an esterification reaction with SAPE (18:0/20:4; Avanti, 850806C) and succinimidyl (NHS) ester-biotin. Triethylamine was added as an alkaline catalyst in the reaction, and the reaction was stirred at room temperature for 12 h in dichloromethane.

**Purification of SAPE-biotin**

SAPE-biotin was purified by Dionex Ultimate 3000 HPLC system using COSMOSIL (Cosmosil) 150 × 4.6 mm 5 μm column. Isocratic mobile phase consists of methanol/H_2_O (90:10, v:v) was used for separation. The purity of product is 91.21%. Structure was confirmed by LC/MS using Q-exactive hybrid-quadrupole-orbitrap mass spectrom­eter (Thermo Fisher Scientific).
